# Using Clinical Trial Simulators to Analyse the Sources of Variance in Clinical Trials of Novel Therapies for Acute Viral Infections

**DOI:** 10.1371/journal.pone.0156622

**Published:** 2016-06-22

**Authors:** Carolin Vegvari, Emilie Cauët, Christoforos Hadjichrysanthou, Emma Lawrence, Gerrit-Jan Weverling, Frank de Wolf, Roy M. Anderson

**Affiliations:** 1 Department of Infectious Disease Epidemiology, School of Public Health, Imperial College London, London, United Kingdom; 2 Janssen Prevention Center, Leiden, The Netherlands; University of Massachusetts, UNITED STATES

## Abstract

**Background:**

About 90% of drugs fail in clinical development. The question is whether trials fail because of insufficient efficacy of the new treatment, or rather because of poor trial design that is unable to detect the true efficacy. The variance of the measured endpoints is a major, largely underestimated source of uncertainty in clinical trial design, particularly in acute viral infections. We use a clinical trial simulator to demonstrate how a thorough consideration of the variability inherent in clinical trials of novel therapies for acute viral infections can improve trial design.

**Methods and Findings:**

We developed a clinical trial simulator to analyse the impact of three different types of variation on the outcome of a challenge study of influenza treatments for infected patients, including individual patient variability in the response to the drug, the variance of the measurement procedure, and the variance of the lower limit of quantification of endpoint measurements. In addition, we investigated the impact of protocol variation on clinical trial outcome. We found that the greatest source of variance was inter-individual variability in the natural course of infection. Running a larger phase II study can save up to $38 million, if an unlikely to succeed phase III trial is avoided. In addition, low-sensitivity viral load assays can lead to falsely negative trial outcomes.

**Conclusions:**

Due to high inter-individual variability in natural infection, the most important variable in clinical trial design for challenge studies of potential novel influenza treatments is the number of participants. 100 participants are preferable over 50. Using more sensitive viral load assays increases the probability of a positive trial outcome, but may in some circumstances lead to false positive outcomes. Clinical trial simulations are powerful tools to identify the most important sources of variance in clinical trials and thereby help improve trial design.

## 1. Introduction

The cost of drug development has increased steadily over the past twenty years. It has been estimated that the total cost of developing a new small molecule drug in North America and Western Europe is now running at an average of $2.558 billion [1, 2]. At the same time, the number of drugs approved per year has declined [3]. Consequently, the cost efficiency of pharmaceutical development has today reached an all-time low [4]. The major part of the total cost is incurred on projects that fail in clinical development [5]. Therefore, reducing the failure rate of clinical trials is arguably the most important step in lowering the cost of drug development [6].

The highest failure rate occurs in phase III clinical trials. The main reason for failure is that the efficacy of a potential novel treatment cannot be demonstrated [3]. It is therefore very important to ascertain if clinical trials fail because the tested treatment is not effective, or because of poor trial design that makes it very difficult to detect the true efficacy of a treatment.

To shed light on these issues, clinical trial simulators can be employed prior to the implementation of a trial, to ascertain what factors in trial design, endpoint definition and variability in measurements might prevent the detection of the true effect. They are rarely used at present, but the cost savings generated by such an approach can be very large, since developing a simulator is relatively cheap by comparison with the recruitment of patients and trial execution.

A successful trial outcome is defined as detecting a statistically significant difference in endpoint measurements between the treatment and the placebo group, in favour of the treatment group. Possible reasons for trial failure (in phase II and III) include too small sample sizes, inadequate endpoints that are unable to detect the impact of a potential treatment, underestimation of individual variation among patients (this includes genetic and environmental variation, and additional demographic factors such as age), underestimation of the complexity of a disease, and measurement variance or experimental error [7, 8]. The concept of variance both in patient characteristics and in endpoint measurements is important to understand why clinical trials fail. There are many sources of variance in clinical trials, and some of them are often unappreciated (Table 1).

**Table 1:** Sources of variances in clinical trials of potential novel therapies for influenza A. Applicable to all acute viral infections.

Individual variability among patients	Measurement variability of viral load assay	Protocol variability
In natural course of infection, In response to treatment	Measurement error, Sensitivity	Number of Measurements, Day of treatment

Measurement and patient variation always reduces the power of the trial and diminishes the reproducibility of results. If there was an unlimited supply of identical patients and measurement techniques were perfect, detecting the true efficacy of a potential treatment would not be a problem. Considering the major sources of variance when planning a clinical trial can obviously increase the robustness of trial design. Robust trial design should allow investigators to reliably reproduce the outcome of a trial [8]. It should also reduce the incidence of false positive or false negative results. Simulating virtual clinical trials before running them in the real world can be used as a tool to assess the robustness of trial design. The variability of different aspects of a clinical trial can be represented as probability functions in the simulation.

In this study, we use a clinical trial simulator to show how the consideration of different sources of variance inherent in clinical trials of possible therapies for acute viral infections can improve trial design and the chances of trial success. We use the example of a phase II clinical trial of a putative new influenza treatment. The setup of challenge studies is less complex than that of phase II and III trials. Consequently, they offer the opportunity to assess several common sources of variance that affect all clinical trials. We investigate the impact of three different types of variance on trial outcome: i) individual patient variability in the natural course of infection and in the response to treatment, ii) the variance of the measurement procedure, and iii) variability of the lower limit of quantification of the endpoint measurements. In addition, we consider how variation in the protocol of the endpoint measurement affects trial outcome.

Although our simulation model and the conclusions we draw from our analysis are specific to infectious disease-related trials, the types of variance we analyse play a role in all clinical trials. Their impact on trial outcome should be analysed using appropriate disease and measurement models prior to trial implementation. The framework of our simulator is adaptable to trials of other diseases by exchanging the disease and measurement models.

## 2. Methods

### Ethics Committee Approval

Written informed consent was obtained from each participant in a form approved by the institutional review boards of the University of Virginia, Charlottesville, and the University of Rochester, Rochester, NY, and subjects were compensated for participation.

### 2.1. Trial simulator design

We have developed a clinical trial simulator (CTS) for challenge studies of novel treatments against influenza A. The purpose of the simulator is to evaluate the probability of running a successful trial, given a specified trial design and varying assumptions about the mode of action and efficacy of the novel treatment. We examine in detail varying assumptions on the error distribution of clinical endpoint measurements. Here we will briefly outline the design and functionality of the simulator. For full details please refer to S1 File.

The trial simulator itself is based on a stochastic, individual-based model of a clinical trial. Each patient in the trial is simulated as an individual entity (object) and runs his/her own instance of the within-host model of influenza A infection. At the same time each patient is part of the overall patient population participating in the trial and the trial group to which he/she has been randomised. For a short description of the within-host model see 2.2. The within-host model is deterministic and the same for all patients. However, the model parameters for each patient are drawn individually from random number distributions, derived based on data from real volunteer challenge studies (for details see 2.3).

During each simulation run the simulated viral load curve over time post infection and post treatment for each patient can be recorded as a reference. In addition, viral load measurements at time points specified by the trial protocol are recorded for each patient. We simulate viral load measurements with qPCR and TCID_50_ assays. As our viral load data consisted only of TCID_50_ measurements and did not include qPCR measurements, we assume the same viral dynamics for qPCR and TCID_50_ curves. This is an oversimplification, and in reality qPCR and TCID_50_ dynamics differ, as [9] have shown. However, here our main purpose is to compare the error distributions of the two assays, rather than their dynamics, and these should not be affected by the dynamics. We explain in the Discussion how this oversimplification may affect our results. We also simulate patient temperature measurements (as a reliable quantifiable symptom measurement) taken at the same time points as viral load measurements. The viral load and temperature measurements can then be used to calculate relevant quantities, such as the area under the viral load curve or reductions in patient temperature, to statistically compare different trial groups.

(The source code of the simulator is included as S3 File CTSCode.zip under the GNU GPLv3 license.)

### 2.2. Within-host model

The within-host model of influenza A infection is a deterministic ordinary differential equation model and has been discussed in depth and validated against patient data in [10].

$$\frac{dT}{dt}=-\beta TV$$

$$\frac{dV}{dt}=r\beta TV-\gamma V$$

Here, *T* is the number of susceptible target cells at time *t*, and *V* is the amount of free virus at time *t*. The parameter β is the infection rate of target cells by free virus, *r* is the rate at which infected cells produce virus, and *γ* is the virus clearance rate. In this model, *γ* subsumes both clearance by the host immune response and non-specific virus decay. Treatment can be incorporated into this model in the following way:

$$\frac{dT}{dt}=-\left(1-\varepsilon_{1}\right)\beta TV$$

$$\frac{dV}{dt}\left(1-\varepsilon_{1}\right)\left(1-\varepsilon_{2}\right)r\beta TV-\left(\gamma -ln\left(1-\varepsilon_{3}\right)\right)V$$

Here *ε*_*i*_ (*i* = 1, 2, 3) is the efficacy of the treatment acting on different stages of the viral life cycle. The efficacy here is defined as the fraction by which a parameter is increased/decreased by the therapy/treatment. Thus, it can be interpreted as the reduction/increase in the rate of the transition in the viral life cycle that the treatment acts on. In particular, efficacy ε_1_ describes the strength with which a potential treatment reduces the infection rate of target cells. Efficacy ε_2_ quantifies the strength with which a potential treatment reduces the virus production rate. Efficacy ε_3_ captures by how much a potential treatment increases the virus clearance rate.

The efficacy in the model is not to be confused with the clinical efficacy of the treatment, defined as the reduction in viral load or the overall reduction in symptoms, that may be measured as an outcome of the trial. In the equations above, the treatment acts on all parts of the virus life-cycle (a realistic assumption for e.g. monoclonal antibodies targeting viral antigens [11, 12]). We assume that the strength with which the treatment acts on each model parameter is the same (*ε*_*1*_ = *ε*_*2*_ = *ε*_*3*_). If the treatment only acts on one model parameter, only the relevant efficacy term is added to the model. This should also approximate the scenario where one mode of action of the treatment is considerably stronger than others. (For example, the treatment mainly acts on the infection rate, but also has minor effects on the virus production and clearance rates). We assume that the efficacy of the treatment is constant, i.e. the half-life of the treatment is longer than the duration of infection (again a realistic assumption for monoclonal antibodies or daily antiviral drug therapy where drug concentrations remains at a high therapeutic level [11]). Treatment can be introduced at different time points post infection in the simulation.

### 2.3. Data used to inform the simulator

The distributions of the within-host model parameters have been estimated from placebo-group data of volunteer challenge studies by Roche using Markov Chain Monte Carlo methods (Table 2). The data were viral load measurements (TCID_50_/ml) of individual participants infected with human influenza A (strain A/Texas/36/91), taken twice daily from day 1 to 3 post infection and once daily thereafter up to day 9. All volunteers in the study were young healthy adults aged 18–27 years (non-smokers). Volunteers were screened for HAI titre against the challenge strain. The parameter estimation methods are described in detail in [13]. We assume a linear relationship between the *log*_*10*_ of the viral load (*V*) and body temperature values, as suggested by patient data analyses and previous publications [14]. A linear regression model for temperature dependent on log10 viral load values was fitted to the data using the lm() function of the R package [15]:

Temperature=36.44°C+0.133°C*log10(V)

**Table 2:** Distribution of within-host model parameters in simulation studies based on estimated parameters from challenge studies. IQR: inter-quartile range.

Infection rate β	Virus production rate r	Virus clearance rate γ
Beta distribution: Shape parameter 1: 0.4858007, Shape parameter 2:862.77162	Beta distribution: Shape parameter 1: 0.3585313, Shape parameter 2: 312.1007775	Gamma distribution: Shape parameter: 1.886947, Rate parameter: 0.5037087
Median: 6.476 x 10–5, IQR: 8.317 x 10–4	Median: 4.1 x 10–4, IQR: 4.543 x 10–4	Median: 2.443, IQR: 2.404

We also assume that body temperature does not fall below the baseline of 36.44°C. Based on dilution series data provided by the Janssen Prevention Center (formerly Crucell), we assume the error of the real-time qPCR assay to be Poisson-distributed. For the TCID_50_ assay we assumed a log-normal distribution of the data points, with a standard deviation of

$$\sqrt{\frac{\text{ln}\left(D_{f}\right)ln2}{n}}$$

where *D*_*f*_ is the dilution factor used in the assay (10 in our analyses) and *n* is the number of wells examined at each dilution step (8 in our analyses) [16].

### 2.4. Simulations

We ran five series of simulations (labelled Experiments 1 to 5 in Table 3) to assess the impact of the following five factors on the probability of trial success: 1) variability among patients in the natural course of infection—assuming that the efficacy of the treatment is the same for all patients; 2) variability among patients in the response to treatment—assuming that the natural course of infection is the same for all patients; 3) variability among patients in the natural course of infection and the response to treatment combined; 4) measurement error of the TCID_50_ and qPCR viral load assays; 5) sensitivity of the different viral load assays.

**Table 3:** Summary of experiments to investigate different sources of variance in clinical trials of novel treatments for influenza A.

Experiment	Source of variance	Simulation setup
1	Individual variability in natural infection	Vary patient parameters of within-host model (see Table 1)
2	Individual variability in response to treatment	Vary efficacy for each patient (draw from normal distribution)
3	Individual variability in natural infection and in response to treatment	Vary patient parameters and efficacy for each patient
4	Distribution of measurement error	Compare error distributions of qPCR and TCID50 assay (Poisson vs. lognormal)
5	Sensitivity of viral load assay	Vary lower limit of quantification of viral load assays
6	Day of treatment	Vary day of treatment (day 1, 2, 3)
7	Frequency of measurements	Vary frequency of viral load measurements (1x, 2x, 3x per day, 2x per day from day 1–3, 1x per day from day 3–9)

In each simulation the trial was set up following a protocol adapted from the original oseltamivir challenge studies conducted by Roche [17]. Volunteers were distributed randomly into the treatment or placebo group. All patients were infected with the same amount of influenza A virus on day 0 and received treatment from day 1 post infection (initial viral load 0.01 TCID_50_/ml). Viral load was measured on days 1, 1.5, 2.0, 2.5, 3, 4, 5, 6, 7, 8 and 9, using qPCR and TCID_50_ assays. Body temperature was measured at the same time points as viral load. In addition, we tested if the day of treatment and the frequency of measurements affected trial outcome (see Experiments 6 and 7).

From these data the area under the curve (AUC) for each patient was calculated from different viral load assays (qPCR, TCID_50_), for the entire simulated viral load curve and for the temperature measurements, using the trapezoid method. The AUCs between the treated and the placebo group were then compared with a Wilcoxon-Mann-Whitney test [18, 19]. Individuals in which the infection did not take off (basic reproductive number for viral replication in host cells *R*_*0*_ < 1, *R*_*0*_ = β*rT*_*0*_/γ) were not excluded from the statistical analysis. In order to examine how the number of volunteers in the study influences the outcome, each experiment was repeated for a total of 50 and 100 participants. Each simulation experiment was repeated 100 times with different random number seeds, to determine the probability that a trial was successful, given the setup and assumptions specified for each experiment. A summary of all experimental setups can be found in Table 2.

#### Experiment 1: Individual variability in natural infection (patient parameters)

For each patient the parameters of the within-host model were drawn from random number distributions as specified in Table 2. The parameter combinations were restricted to values that gave a basic reproductive number *R*_*0*_ of less than 40 [20]. We restricted the upper boundary of *R*_*0*_ to avoid numerical errors that very high values of *R*_*0*_ caused in the integration of the within-host model equation system. These errors occurred when the value of *R*_*0*_ was very (and probably unrealistically) high. We chose 40 as an upper limit, because in our analysis and in most previous studies, e.g. [20], the value of *R*_*0*_ values was not greater than 40. The assumed efficacy of the treatment was fixed in each simulation setup. However, we varied the efficacy in a series of different simulations from 0 to 95%, to see how likely the trial is to be successful over a range of possible efficacies. This consideration is especially relevant to novel immunotherapies that may not be 100% effective, but still cause a significant reduction in AUC compared to placebo.

We tested different assumptions on the mode of action of the novel treatment in different simulation setups. We assumed that the treatment would act exclusively either on the infection rate of susceptible cells (β), the virus production rate (*r*), or the virus clearance rate (γ), or on all model parameters. We assume a lower limit of quantification (LLOQ) of 3.33 log_10_ viral cDNA copies/ml for the qPCR assay [21], and 2.0 log_10_ units for the TCID_50_ assay.

#### Experiment 2: Individual variability in response to treatment (efficacy of treatment)

The within-host model parameters were the same for each patient (median of estimates). The efficacy of the treatment was individual-dependent, i.e. it was drawn from a truncated normal distribution (ranging from 0–1) for each patient. We varied the mean efficacy from 0–95% (0.0, 0.1, 0.2, 0.3, 0.4, 0.5, 0.6, 0.7, 0.8, 0.9, and 0.95) and used different standard deviations in different simulation runs (0.001, 0.01, 0.1, 0.2, 0.5, and 1.0). As in Experiment 1, we varied the mode of action of the new treatment in different simulation runs. We set the LLOQ of the qPCR assay to 3.33 log_10_ units and that of the TCID_50_ assay to 2.0 log_10_ units.

#### Experiment 3: Individual variability in natural infection and response to treatment

Here we drew both the model parameters and the treatment efficacy for each patient from random number distributions, as detailed for Experiments 1 and 2, respectively. The error distributions and LLOQs of the viral load assays stayed the same as in Experiment 1.

#### Experiment 4: Measurement error (TCID50, qPCR)

In this set of experiments we drew the parameters of the within-host model from random number distributions for each patient and kept the efficacy constant for all patients within the same simulation run (as in Experiment 1). The error distributions and sensitivities of the viral load assays were set as in Experiment 1.

#### Experiment 5: Assay sensitivity (lower limit of quantification)

The protocol for Experiment 5 was the same as for Experiment 1, but here we examined different values for the LLOQ of the qPCR and the TCID_50_ assays (assumed LLOQs for qPCR: 2.0 log_10_ units, 2.5 log_10_, 3.33 log_10_, 4.3 log_10_ units; assumed LLOQs for TCID_50_: 0.0 log_10_, 1.0 log_10_, 2.0 log_10_, 2.5 log_10_).

#### Experiment 6: Day of treatment

We followed the same setup as in Experiment 1, but simulated treatment from day 2 and day 3, instead of day 1.

#### Experiment 7: Frequency of measurements

We followed the same setup as in Experiment 1, but used different measurement protocols for viral load and symptoms: one measurement per day, two measurements per day, or three measurements per day.

## 3. Results

### 3.1. Individual variability (Experiments 1, 2 and 3)

#### Experiment 1: Individual variability in natural infection

**Fig 1:**
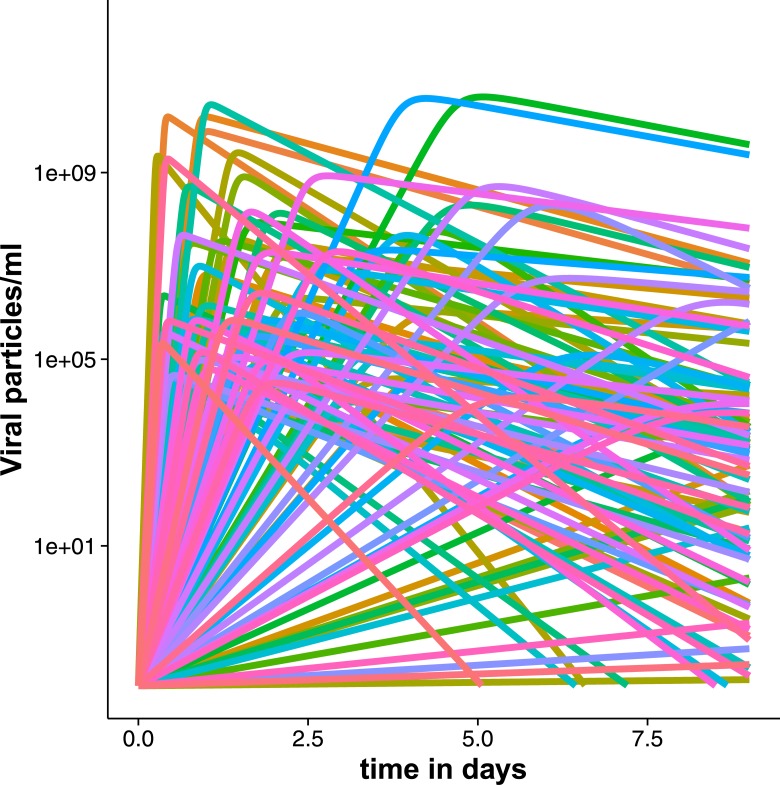
Viral load curves over time of 50 (a) and 100 (b) patients. If there are less curves than simulated patients, the infection did not take off in the remaining number of patients (*R*_*0*_ < 1) (curve is a flat line across the x-axis). x-axis: time in days, y-axis: viral load in particles per ml.

**Fig 2:**
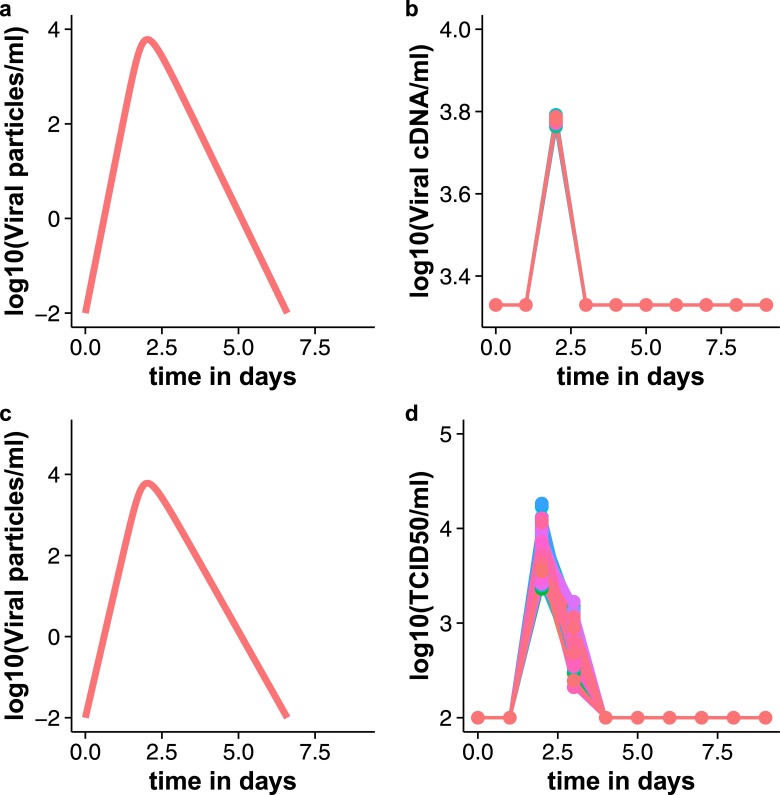
Variance of viral load measurements. For this figure we simulated influenza virus infection in 1000 patients that all had the same natural course of infection. For each of the patients, viral load measurements were generated, simulating qPCR (upper row) and TCID_50_ assays (lower row). The variance of the TCID_50_ assay (lognormal distribution) is greater than that of the qPCR assay (Poisson distribution). The variance of the viral load assays is small compared to the variation in natural infection among patients. Upper row: qPCR measurements, unit: viral cDNA/ml. Lower row: TCID_50_ measurements, unit TCID_50_/ml. a, c: True (simulated) viral load curves of 1000 patients with same course of natural infection. b, d: simulated measurements of 1000 patients with the same natural course of infection. b: lower limit of quantification of the PCR assay was 3.33 log_10_ cDNA/ml (2137 cDNA/ml), d: lower limit of quantification of TCID_50_ assay was 2 log_10_ TCID_50_/ml (100 TCID_50_/ml).

**Fig 3:**
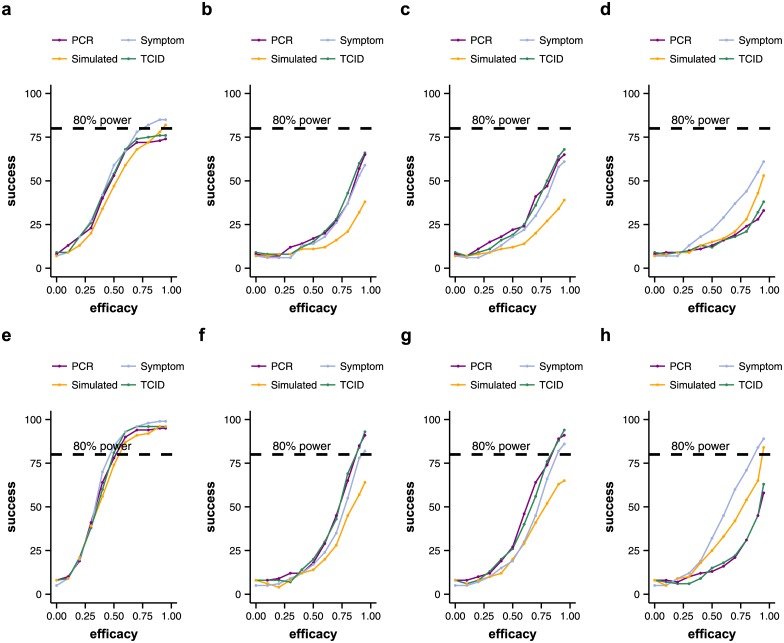
Experiment 1: Individual variability in natural infection. The plots show the number of successful trials out of 100 simulated trial runs (y-axis) for different assumed efficacies of the treatment (x-axis). All 100 iterations for each assumed efficacy value had exactly the same setup and differed only in the random number seed. The number of successful trials out of 100 runs can be interpreted as the power of the trial. The power of the trial depends on the mechanism of action of the treatment and the number of patients in the trial. As individual variability in natural infection is large, trials with 50 patients do not reach a power of 80%, even if the assumed efficacy of the treatment is high (90+%). Coloured lines show the number of successful trials out of 100 runs depending on efficacy for different endpoint measurements (PCR: viral load AUC measured with qPCR; Symptom: temperature AUC; TCID: viral load AUC measured with TCID_50_; Simulated: viral load AUC of the simulated viral load AUC). Upper row: trials with 50 patients. Lower row: trials with 100 patients. a, e: treatment acts on all stages of the virus life cycle/model parameters. b, f: treatment acts on the infection rate. c, g: treatment acts on the virus production rate. d, h: treatment acts on the virus clearance rate.

The variability in the course of natural infection is high, even though the individual patient parameters on which the simulations were based were estimated from a very homogenous demographic sample (Fig 1). The range of variability in the simulated course of natural infection is similar to predictions found in earlier modelling studies [22]. The variability among patients is large, compared to the variance of the measurement error of the viral load assays (Figs 2 and 3). The duration of infection ranged from 0 days (infection did not take off, *R*_*0*_<1) to more than 8 days (duration of measurement period). Peak viral load varied between 0 (infection did not take off, *R*_*0*_<1) to 3 x 10^6^. Moreover, the timing of peak viral load varied considerably from less than 1 day after infection to day 7.

**Fig 4:**
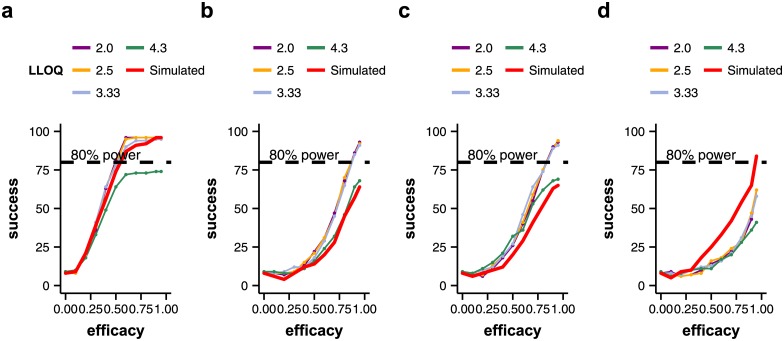
Experiment 5: Sensitivity of viral load assay (qPCR). Plots show the number of successful trials out of 100 runs (y-axis) over the assumed mean efficacy of treatment (x-axis). The probability of success corresponds to the power of the trial. The parameters determining the course of natural infection were drawn from the same random number distributions for each patient as explained in the main text. The efficacy for each patient (response) was fixed to the same value for each patient in each run. Thin coloured lines show the power of the trial dependent on the efficacy of the treatment for qPCR viral load assays assuming different lower limits of quantification. The bold red line shows the power of the trial dependent on the efficacy of the treatment, if the simulated viral load curve is considered. Very insensitive assays can greatly reduce the power of a trial, especially in potent drugs that act on several stages of the virus life cycle (a). Conversely, if the treatment acts on the infection rate (b) or the virus production rate (c), very sensitive viral load assays tend to give false positive results. Trials with 100 patients. a: treatment acts on all model parameters. b: treatment acts on the infection rate. c: treatment acts on the virus production rate. d: treatment acts on the virus clearance rate.

We found that the assumed mechanism of action of the tested treatment had a great impact on trial outcome. For treatments that act on viral growth parameters (infection rate, virus production rate), both viral load assays and body temperature tend to overestimate the efficacy of the treatment. They may indicate that the difference in AUC between the treated and untreated groups is statistically significant, when the difference in AUC between the treated and placebo groups of the simulated viral load curves is not (Fig 4). By simulated viral load curves we mean the entire simulated course of infection of each patient from which measurements are taken. These curves cannot be known in reality, as only measured values can be observed.

For treatments that act on the virus clearance rate, both qPCR and TCID_50_ assays tend to underestimate the efficacy of the tested treatment and give false negatives, compared to the outcome of the simulated viral load curves (Fig 4). Contrarily, if body temperature AUC is used as a clinical end point, the efficacy of the tested treatment tends to be overestimated. Treatments that act on all aspects of the viral life cycle, have the highest probability of leading to a successful trial outcome. They also have the highest agreement on outcome between viral load assays, symptom measurements and simulated viral load curves (Fig 4).

As expected, the probability of running a successful trial (power of the trial) is higher with 100 participants than with only 50 (Fig 4). Even with a treatment efficacy of 95%, no trial with only 50 patients in Experiment 1 had a power of 80% or more according to viral load measurements (Fig 4). In contrast, with 100 patients and assuming a high efficacy of the treatment (at least 90%), almost all trials had a power of 80% or greater (even if these include some false positive results). The only exceptions were trials of treatments that act on the virus clearance rate, which never reached a power of 80%. This indicates that the impact of potential novel treatments acting only or mainly on the virus clearance rate may be difficult to detect with this phase II trial protocol. More detailed within-host models of influenza infection may be better able to confirm this prediction [23].

#### Experiment 2: Individual variability in response to treatment

Compared to the impact of individual patient variability in the natural course of infection, patient variability in the response to treatment had a negligible effect on trial outcome (Figures A-D in S2 File). However, the setup of Experiment 2 was highly artificial (assuming that all patients had exactly the same natural course of infection and only differed in their response to treatment). Therefore, we considered a more realistic scenario (patients varied in the natural course of infection and in their response to treatment) in Experiment 3.

#### Experiment 3: Individual variability in natural infection and response to treatment

If individual variability in response to treatment was low (10% of the population mean of the efficacy or less), compared to individual variability in the course of natural infection, it did not affect trial outcome (Figures E-H in S2 File). Contrarily, if individual variability in response to treatment was similar to or greater than individual variability in the course of natural infection, the power of a clinical trial could be greatly reduced (to less than 50%) (Figures E-H in S2 File). The explanation is that even if the mean efficacy of the treatment was high, there were many patients who would not respond as well to treatment, so that the measureable impact on infection in these patients was lower. Apart from these additional results, the same observations as in Experiment 1 apply to Experiment 3.

### 3.2. Variance of viral load measurements

#### Experiment 4: Measurement error (TCID50, qPCR)

Although the qPCR and TCID_50_ assays had different error distributions, they had almost the same ability to detect a statistically significant difference between treated and untreated groups. They also had the same tendency to detect false positive or negative outcomes (Fig 4). The impact on clinical trial outcome of the measurement error of the viral load assay may not have been as great as that of the sensitivity of the assay.

### 3.3. Sensitivity of the viral load assay

#### Experiment 5: Assay sensitivity (lower limit of quantification, LLOQ)

**Fig 5:**
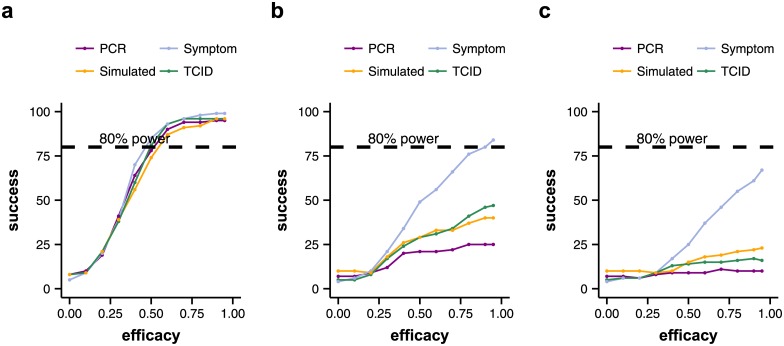
Experiment 6: Day of Treatment. Treatment acts on all model parameters. Plots show the number of successful trials out of 100 runs (y-axis) over the assumed mean efficacy of treatment (x-axis). The probability of success corresponds to the power of the trial. The parameters determining the course of natural infection were drawn from the same random number distributions for each patient as explained in the main text. The efficacy for each patient (response) was fixed to the same value for each patient in each run. The later treatment is given, the lower the power of the trial. a: treatment on day 1; b: treatment on day 2; c: treatment on day 3. Coloured lines show power of the trial depending on efficacy for different endpoint measurements (PCR: viral load AUC measured with qPCR; Symptom: temperature AUC; TCID: viral load AUC measured with TCID_50_; True: viral load AUC of the simulated viral load AUC).

Low-sensitivity assays can reduce the ability of a trial to detect a statistically significant difference between treated and untreated groups. When the treatment acted on the viral clearance rate or on all model parameters, a very insensitive assay (qPCR, LLOQ 4.3 log_10_ units) considerably reduced the power of the trial (Fig 5). When the treatment acted on the infection rate or the virus production rate, however, more sensitive qPCR assays resulted in more false positive trial outcomes at high efficacy, compared to differences in simulated viral load AUCs.

TCID_50_ assays tend to be more sensitive than most frequently used qPCR assays when it comes to influenza virus quantification [24, 25]. Consequently, variability in the lower limit of quantification only had little effect on trial outcome in our analysis (Figures I-J in S2 File).

### 3.4. Trial design

#### Experiment 6: Day of treatment

When treatment was given after the time of peak viral load (day 2 or day 3), the probability to detect a statistically significant difference between treated and untreated groups was considerably reduced, compared to when treatment was given before the peak (day 1) (Fig 5). Both viral load assays and symptom (temperature) measurements tended to give false positive trial outcomes for all modes of action of treatments, apart from those acting only on the virus clearance rate (Figures K-N in S2 File).

#### Experiment 7: Frequency of measurements

Increasing the frequency of viral load measurements beyond 1 per day did not improve the power of the trial. In other words, it did not increase the probability to find a statistically significant difference between treated and untreated groups in viral load or symptom AUCs (Figures O-R in S2 File).

## 4. Discussion

Our analysis demonstrates that the biggest source of variance in a challenge study of treatments for influenza A infection is the individual patient variability in the natural course of infection, as measured by changes in viral load over time. This implies that underestimating the population variability in the course of natural infection may be the main reason for trial failure. Trial failure here is defined as failure to detect a statistically significant difference in endpoint measurements between treatment and placebo groups, when the treatment is actually effective.

Including more participants in a trial will reduce the probability of chance effects leading to trial failure or false positive trial outcomes. Running a phase II trial with 100 rather than 50 Patients can lead to considerable cost savings. Assuming an average cost per patient per trial of $40,000 for phase II trials and $42,000 for phase III trials and an average number of 1,000 patients in a phase III trial for potential novel influenza treatments [26], the cost for an unsuccessful phase III trial after a falsely positive phase II trial are $42 million. The cost of a phase II trial with 100 patients are $4 million. So the potential savings allowing for greater variation among patients are $38 million.

In the artificial scenario where all patients had exactly the same course of natural infection (Experiment 2), the power of the trial to detect a statistically significant difference between groups was almost always 100%. This was observed regardless of trial size and other variables. In contrast, when patient variability as estimated from a demographically homogenous sample of volunteers infected with influenza A was considered (Experiment 1), the power of the trial was greatly reduced.

Individual variability in the response to treatment can further reduce the power of the trial (Experiment 3). Although reliable information on population variation in the response to treatment is unlikely to be available at the beginning of a phase II trial for a novel treatment, generating this information should be one of the goals of the trial. This can be done by assessing the variability in infection in the treated group and comparing it with the variability in the placebo group. Overall our results suggest that any novel influenza treatment will need a high efficacy to achieve a statistically significant difference in viral load AUC compared to placebo. High efficacy of the novel treatment may also prevent the emergence of resistance, which has been shown to be a problem for existing anti-influenza treatments [27, 28].

In our simulations, the assumed mechanism of action of the treatment had a major impact on the power of the trial. Unsurprisingly, trials of treatments that act on all stages of the virus life cycle tend to have the highest power. Trials of treatments that act on the virus clearance rate tended to have the lowest power in our analysis. The reason is that in our analysis such treatments mainly increased the viral load decay slope without greatly affecting the area under the viral load curve. Using a multilevel model, Heldt et al. [23] found that treatments that act on virus production are most effective in reducing viral load, whereas treatments that reduce the infection rate primarily delayed the course of infection. In our simulations, trials of treatments acting on virus production vs. those of treatments acting on the infection rate tended to have similar power, although the success rate of trials for treatments reducing virus production may be slightly higher.

As all participants in volunteer challenge studies are routinely tested for pre-existing HI-titres against the challenge strain, pre-existing humoral immunity cannot be the cause of the high observed population variability in the course of natural infection [29]. Several recent studies have found a correlation between pre-existing cellular immunity to influenza A and disease protection [29–31]. Following these observations and our analyses, it may be advisable to incorporate screening for pre-existing cellular immunity against influenza A into the selection process of participants in challenge studies, to increase the chance of detecting a statistically significant difference between treatment and placebo groups and to better be able to characterise the treatment effect under controlled conditions [11, 32].

On the other hand, a highly selected sample of the population, screened for both pre-existing humoral and cellular immunity, may not be representative of the eventual target population of the treatment [33]. Larger and more inclusive trials need to be run subsequently to assess the effectiveness of the treatment in the general population. Our analyses indicate that detecting the effect of the treatment may be difficult even in a very demographically homogeneous population.

Besides differences in infection history, genetic differences among patients play an important role in overall population variability in natural infection and response to treatment [34]. [11, 32]. More broadly, it is well known that the course of viral infections within mammalian hosts is strongly influenced by the genetic background of the host and this is very difficult to control for at present in human volunteer studies given limited knowledge of the genetic determinants of the severity of influenza A infection [35].

Efforts to uncover human genetic variation that underlies severe influenza infection are ongoing [36, 37]. Additionally, inbred mouse strains can be used to assess the impact of host genetic variability on variation in infection and to identify genetic variants that influence the course and severity of infection [38]. Genetic markers that are known to affect the course and severity of influenza infection can be used in screening of volunteers in challenge studies or for post-hoc analysis of clinical trial data. But at present, patient variability in infection must be recognised and its impact must guide trial design.

Our results imply that both qPCR and TCID_50_ assays perform equally well at detecting statistically significant differences between treated and untreated groups, when viral load AUC is used as an endpoint. However, we also found that the sensitivity of the viral load assay can greatly influence trial outcome, and the effect may depend on the mechanism of action of the tested treatment. While small differences in assay sensitivity did not change trial outcome (e.g. lower limit of quantification 2.0 vs. 2.5 log_10_ units), a very high lower limit of quantification may greatly reduce the power of a trial (LLOQ 4.3 log_10_ units). Conversely, for some treatments, a more sensitive viral load assay may increase the probability of falsely detecting a positive trial outcome. The choice of the viral load assay should, therefore, be based on knowledge about the mechanism of action of the tested treatment.

Petrie et al. [9] showed that viral titre dynamics differ when measured with RT-PCR or the TCID_50_ assay, because RT-PCR measures all viral RNA, including non-functional virus, whereas TCID_50_ only measures infectious virus. Therefore investigators should choose the assay depending on whether they are more interested in the clearance of infectious virus or total virus.

We found that the later a treatment is given, the lower the probability to detect a statistically significant effect on infection. Our results agree with those from [28], where the authors found that treatment efficacy declines right from the time of inoculation. This may prove problematic for intention-to-treat trials, because patients usually present at clinics when the time of peak viral load has already passed [33]. Moreover, subjects will start treatment at different time points after infection. In these cases, viral load or symptom AUC may not be a suitable endpoint measurement. Changes in the slope of viral load and symptom decline or time to resolution of illness/first negative viral load measurement may be more sensitive endpoints in studies, where treatment is given late in the course of infection [39, 40].

Increasing the frequency of viral load measurements to more than one per day did not improve the power of the trial in our analysis. The reason is that, although more measured data points provide more accurate information on the shape of the viral load curve and hence the AUC, the measurement protocol equally affects both the treatment and placebo group. Moreover, we used a non-parametric statistical test to compare the two groups. Consequently, more accurate information on the magnitude of the AUC may not affect the outcome of the test, as long as the ranking of AUCs across patients stays the same. Alternatively, this finding may be an artefact of the simple model used to generate the viral load curves. Additional sampling points could be important if the viral load curve, and therefore the shape of the AUC, were simulated with more complex models that include, for example, explicit representations of the immune response or resistant virus.

To summarise, our results suggest that the following aspects are the most crucial in the design of volunteer challenge studies to test potential novel treatments of influenza A:

1. The population variability in the course of natural infection is large and should not be underestimated. Additional screening steps may be introduced to make sure that participants in a volunteer challenge study form as homogenous a sample as possible, to increase the chance of detecting a statistically significant difference between treatment and placebo in a challenge study. As the variability in the eventual target population will be much larger, one cannot simply extrapolate results concerning trial success from a challenge study to a phase III trial. However, well controlled conditions in challenge studies can help in the learning phase to better characterise the effect of a novel treatment. This will also help in the planning of more robust phase IIb/III trials.
2. A trial with 100 participants is preferable over a trial with only 50 participants, if—assuming a high efficacy of the treatment—the trial should have a power of at least 80%. For treatments with a lower efficacy, even more participants would be required.
3. The mechanism of action of the potential novel treatment determines how it will affect the shape of the viral load curve over time and hence the course of infection. Ideally the precise mechanism of action of the novel treatment should be known prior to the start of a clinical trial.
4. If the treatment acts on the virus clearance rate or on several stages of the influenza virus life cycle, a sensitive viral load assay should be used. If the treatment acts primarily on the infection rate or the virus production rate, a less sensitive assay may avoid false positive trial outcomes.
5. For some types of treatments, we observed a tendency of both measured viral load and symptom endpoints to signal a statistically significant difference between treated and placebo groups, even if the simulated viral load AUCs between the groups did not differ significantly. This may indicate a tendency for phase II trials to overestimate the efficacy of a novel influenza treatment, which may lead to disappointment in phase III studies.

## 5. Conclusion

A quantitative approach to biological and clinical endpoint measurements based on data analysis and clinical trial simulators can help to identify the major sources of variance in clinical trial design. Thus, it opens the possibility to make trial design more robust by controlling for variation and minimizing uncertainty at all stages of the trial. Although our analysis here specifically considered a phase II trial of potential new influenza A treatments, the same sources of variance affect any clinical trial (individual variation, measurement error, measurement sensitivity, and protocol variation). Clinical trial simulators are valuable tools for analysing and improving trial design prior to trial implementation. The cost of constructing the simulator is many orders of magnitude less than the cost of running a trail. Simulators help to minimise the likelihood of failure due to poor design, poor measurement choice or inadequate sample size. Trial simulators can help to maximise the chances of trial success and to reduce the costs of drug development.

## Supporting Information

S1 File (DOCX)

S2 File (DOCX)

S3 File Clinical Trial Simulator code files. S3 does not contain any information that would allow the identification of human subjects.
(ZIP)
