# Supplementary material for: Using Clinical Trial Simulators to Analyse the Sources of Variance in Clinical Trials of Novel Therapies for Acute Viral Infections
Source: PLoS One. 2016 Jun 22;11(6):e0156622. doi: 10.1371/journal.pone.0156622 (PMC4917234; doi:10.1371/journal.pone.0156622)
Supplement: S1 File — (DOCX) [file pone.0156622.s001.docx]

**S1 – Description of the Clinical Trial Simulator**

*The description of the simulator follows guidelines adapted from the ODD (Overview, Design Concepts, Details) protocol for individual- and agent-based models suggested by (1), and updated by (2).*

**Overview**

Purpose: We have developed a clinical trial simulator (CTS) for phase II and III trials of novel treatments against influenza A. The purpose of the simulator is to evaluate the probability of running a successful trial, given a specified trial design, varying assumptions about the mode of action and efficacy of a novel treatment, and assumptions on the error distribution of clinical endpoint measurements. Trial success is defined as a statistically significant outcome in favour of the tested treatment.

Entities and variables: The trial simulator consists of a stochastic and a deterministic part. The functional modules of the trial simulator are shown in Fig S1.1. The clinical trial itself is represented by a stochastic, individual-based model. A set number of patients can be allocated randomly or according to specified criteria to a given number of trial groups. Patient characteristics and parameters are set at the beginning of each simulation according to specified random number distributions. In the volunteer challenge study that we consider as part of this paper, the only patient-specific characteristics we simulate are the parameters of the within-host model of influenza infection and treatment. The within-host model is a deterministic, ordinary-differential equation model that describes the development of viral load within an infected person over time. We explain the within-host model and its parameters in more detail below.

Other model parameters that we vary are the efficacy of the treatment and the lower limit of quantification of the viral load assay. The efficacy of the treatment is a model parameter that expresses the quantitative change in the stage of the viral life cycle on which the treatment acts. It is not to be confused with the *clinical efficacy* of the treatment, defined usually as the reduction in viral load or the overall reduction in symptoms in the treatment group relative to the placebo group. Thus, while the efficacy as a model parameter is an input variable with a biological meaning, we can measure the clinical efficacy as an outcome of the trial.

Process overview and scheduling: At the beginning of each simulation run of the challenge study, a given number of patients are generated (patient population), and the parameter values for each patient are drawn randomly from specified distributions. Then the patients are distributed at random to either the treatment or the placebo group. Virus inoculation is simulated at day 0, i.e. this is when the within-host model of infection starts in all patients. At this time there is no treatment in any of the patients. Treatment of a specified efficacy and placebo are given on day 1 (or day 2, or day 3, depending on the simulation) after infection.

**Trial**

**Setup**

**Input**

**Population**

**Recorder**

**Group**

**Patient**

**Within-Host**

**Model**

**of Disease Progression**

**Statistics**

**Module**

**Plotting**

**Module**

**Output**

**Meta-Recorder**

**Fig S1.1: Diagram of Clinical Trial Simulator modules.** The trial simulator is implemented as modular program coded in C++. Different modules represent different entities that play a role in the trial, but also functionality to record and analyse the results. Input: takes input parameters for the simulation. Trial setup: functions to design the trial according to protocol. Population: patient population. Group: different treatment groups in the trial. Patient: patients belong to the background population and are allocated to treatment groups. Within-host model: each patient entity has its own representation of the within-host model. Recorder: records trial observations. Plotting module: plots individual patient viral load curves, trial outcome and summary output. Statistics module: functions for the statistical analysis of the trial data. Meta-recorder: records summary data from many trials run with different random number seeds and/or different parameter settings. Output: outputs data plots and summary statistics. The plotting module of the simulator uses the gnuplot-iostream.h file by Dan Stahlke available at <http://www.stahlke.org/dan/gnuplot-iostream> to link the C++ code to the plotting programme gnuplot.

In the placebo group nothing happens, and the infection continues. In the treatment group the patient parameters are multiplied with an “efficacy term” that represents the effect of the treatment, and the within-host model continues with the new parameter values (for details see below).

The viral load is measured at time points specified in the trial protocol (e.g. once per day on days 1, 2, …, 8 post infection). For more details on how we model the measurement procedure refer to the submodels section. Throughout the infection the temperature curve of each patient develops in correlation with the viral load curve (for details see submodels). Temperature is measured at the same time points as viral load. In addition, the “true” viral load curve is recorded for each patient as a reference. A simulation run ends, when the last viral load measurement has been taken. Then, the area under the curve (AUC) of the viral load is calculated for each patient using the trapezoid method. The Wilcoxon-Mann-Whitney test is used to statistically compare the AUCs between the treatment and the placebo group. Each simulation is repeated with different random number seeds 100 times.

**Design concepts**

Basic principles: The premise of the individual-based model of the trial simulator is that the results of many stochastic processes in the clinical trial can affect the overall outcome of the trial. The uncertainties at subsequent steps in the trial are multiplicative (the probabilities of events multiply at each step). The higher the uncertainty associated with each of these processes the higher the uncertainty about the trial outcome as a whole will be.

We chose a deterministic model of influenza A infection, because preliminary analyses have shown that the number of virus particles produced during an infection is so large that stochastic effects on the viral load curve are negligible. The model is an ordinary differential equation system that represents the rate of change over time in the measured virus concentration and the change in the number of susceptible target cells.

Stochasticity: The random processes in the trial model are the choice of patient parameters at the beginning of the simulation and the error for the viral load measurement at each time point. As the shape of the “true” viral load curve for each patient depends on the patient parameters, the position of the measured viral load points in relationship to the true curve is another random factor. We do not consider drop-outs or missing data in this set of analyses.

Observations: The data observed in this simulation model are the viral load measurements with different assays and the temperature measurements which are taken at time points specified in the trial setup, and, as reference values, the true viral load and temperature curves for each patient.

**Details**

Initialisation: At the start of each simulation the total number of patients participating in the trial is set, and the patients are distributed evenly and randomly across the treated and placebo groups. Patient parameters are drawn at random from distributions that are based on real-world data from actual challenge studies (Table S1.1). The parameter combinations are restricted to values that give a basic reproductive number R_0_ of less than 40. The starting viral load for all patients is set to 0.01 TCID_50_/ml, as it has been shown that the initial viral load has a negligible effect on the course of infection, unless it is very large (3).

Table S1.1: Distribution of within-host model parameters in simulation studies based on estimated parameters from real challenge studies.

| Infection rate β | Virus production rate r | Virus clearance rate γ |
| --- | --- | --- |
| Beta distribution  Shape parameter 1: 0.4858007  Shape parameter 2:  862.77162 | Beta distribution  Shape parameter 1:  0.3585313  Shape parameter 2:  312.1007775 | Gamma distribution  Shape parameter:  1.886947  Rate parameter:  0.5037087 |

Input data: The viral load data used to estimate the distributions of the patient parameters were taken from the placebo groups of the original oseltamivir volunteer challenge studies conducted by Roche. The data were viral load measurements (TCID_50_/ml) of individual participants infected with human influenza A (strain A/Texas/36/91) taken from day 1 post infection to day 9. The parameters for each participant were estimated by MCMC (4). The population distributions of the parameters were assessed from Cullen-Frey graphs (R function descdist from package fitdistrplus (5)) and then fitting the most likely distribution to the median parameter estimates (also with the fitdistrplus package). The initial number of susceptible target cells was set to 4 x 10^8^, which is an estimate of the number of epithelial cells in the human upper respiratory tract (6).

Submodels:

The within-host model has been presented in full in the Methods section of the main manuscript.

**References**

1. Grimm V, Berger U, Bastiansen F, Eliassen S, Ginot V, Giske J, et al. A standard protocol for describing individual-based and agent-based models. Ecological Modelling. 2006;198(1-2):115-26.

2. Grimm V, Berger U, DeAngelis DL, Polhill JG, Giske J, Railsback SF. The ODD protocol: A review and first update. Ecological Modelling. 2010;221(23):2760-8.

3. Hancioglu B, Swigon D, Clermont G. A dynamical model of human immune response to influenza A virus infection. Journal of theoretical biology. 2007;246(1):70-86.

4. Vegvari C, Hadjichrysanthou C, Cauët E, Lawrence E, Wolf FD, Anderson R. Measuring the impact of novel therapies for acute viral infections. EBioMedicine. in review.

5. Delignette-Muller M, Dutang C. fitdistrplus: An R Package for Fitting Distributions. Journal of Statistical Software. 2015;64(4):1-34.

6. Baccam P, Beauchemin C, Macken CA, Hayden FG, Perelson AS. Kinetics of influenza A virus infection in humans. Journal of virology. 2006;80(15):7590-9.
