# Supplementary material for: Using Clinical Trial Simulators to Analyse the Sources of Variance in Clinical Trials of Novel Therapies for Acute Viral Infections
Source: PLoS One. 2016 Jun 22;11(6):e0156622. doi: 10.1371/journal.pone.0156622 (PMC4917234; doi:10.1371/journal.pone.0156622)
Supplement: S2 File — (DOCX) [file pone.0156622.s002.docx]

**S2 – Supplementary Figures**

a

b

c

d

e

f

g

h

**
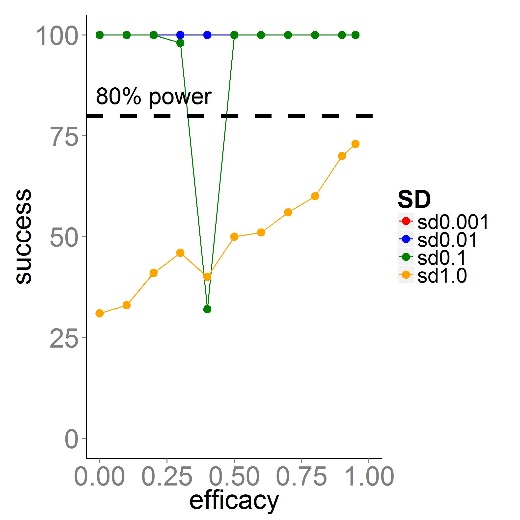

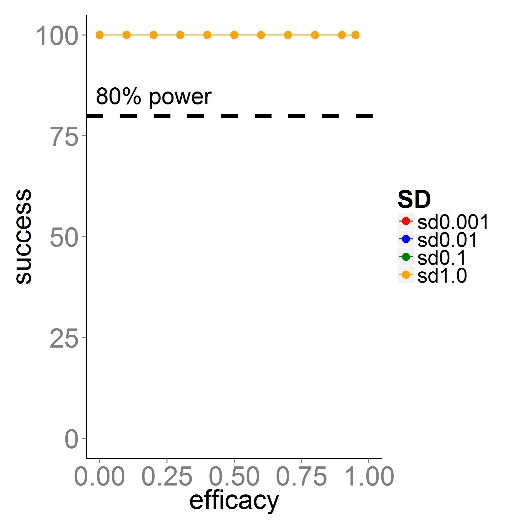

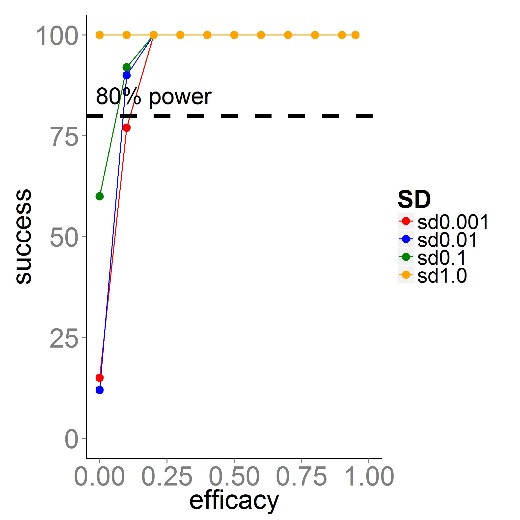

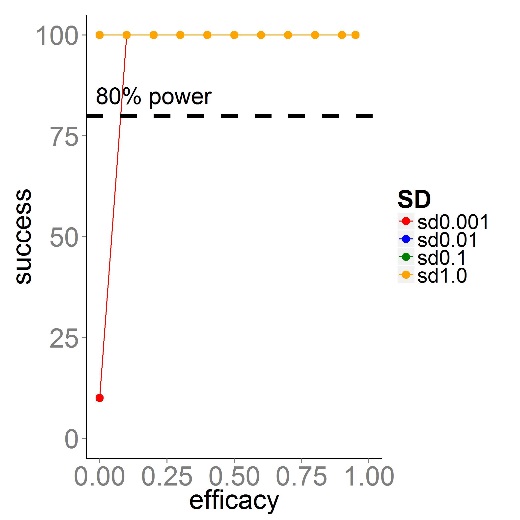
**

Fig A: Experiment 2: Individual variability in response to treatment. Treatment acts on all model parameters. Plots show the number of successful trials out of 100 runs (y-axis) over the assumed mean efficacy of treatment (x-axis). The probability of success corresponds to the power of the trial. All patients had exactly the same course of natural infection. The efficacy for each patient (response) was drawn from a normal distribution with standard deviations as shown in the plot legends. Upper row: trials with 50 patients. Lower row: trials with 100 patients. a, e: endpoint viral load area under the curve (AUC) from qPCR measurements. b, f: endpoint AUC from TCID_50_ measurements. c, g: endpoint AUC of simulated viral load. d, h: endpoint temperature AUC.

**
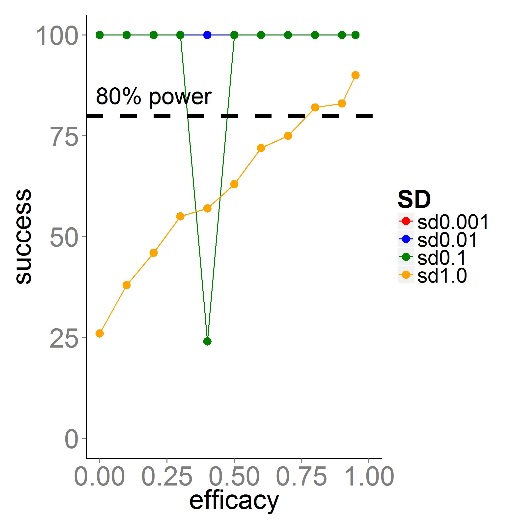

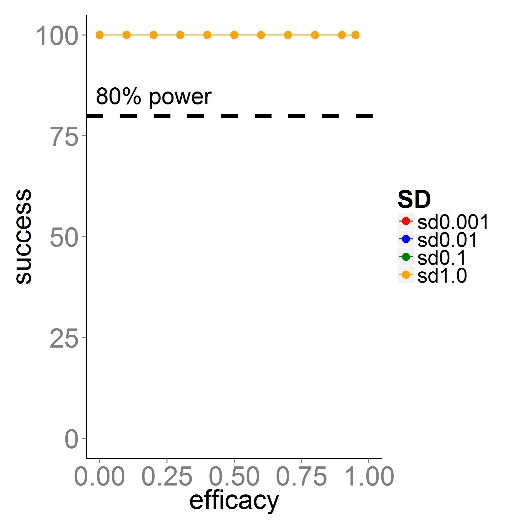

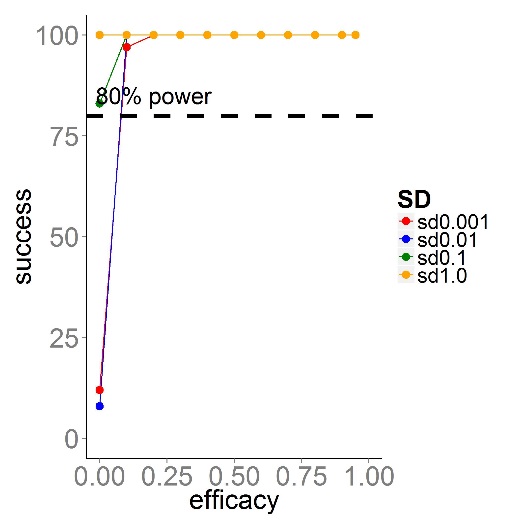

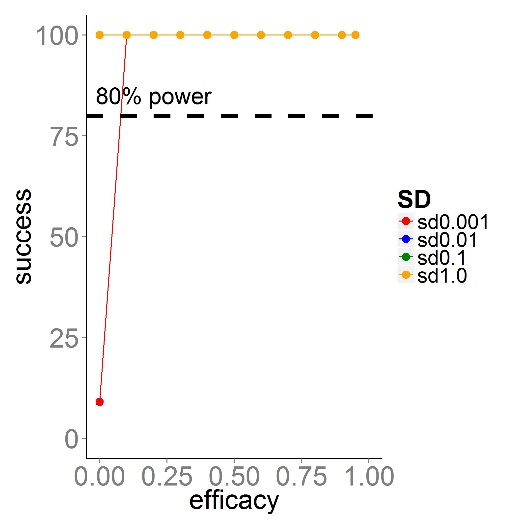
**


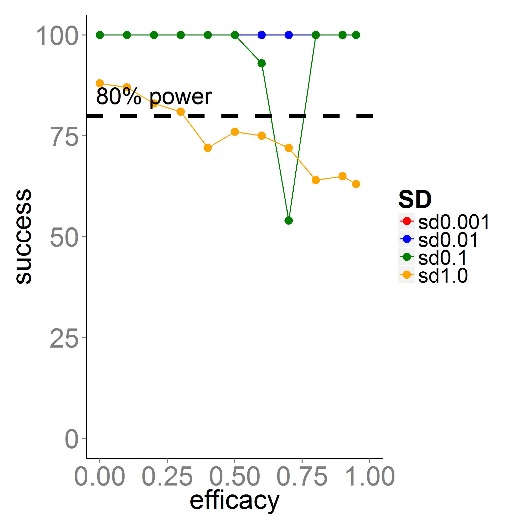

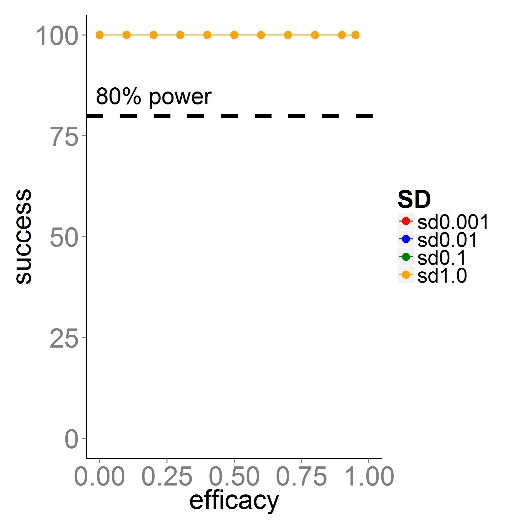

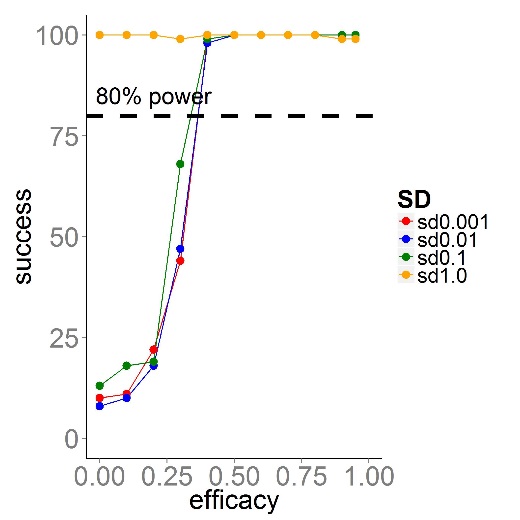

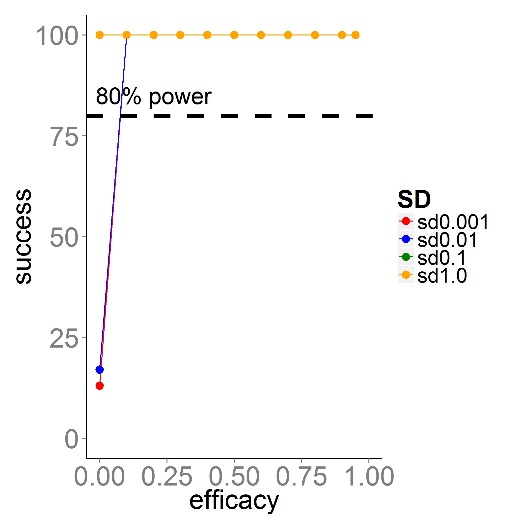


h

g

f

e

d

c

b

a

Fig B: Experiment 2: Individual variability in response to treatment. Treatment acts on infection rate. Plots show the number of successful trials out of 100 runs (y-axis) over the assumed mean efficacy of treatment (x-axis). The probability of success corresponds to the power of the trial. All patients had exactly the same course of natural infection. The efficacy for each patient (response) was drawn from a normal distribution with standard deviations as shown in the plot legends. Upper row: trials with 50 patients. Lower row: trials with 100 patients. a, e: endpoint viral load area under the curve (AUC) from qPCR measurements. b, f: endpoint AUC from TCID_50_ measurements. c, g: endpoint AUC of simulated viral load. d, h: endpoint temperature AUC.


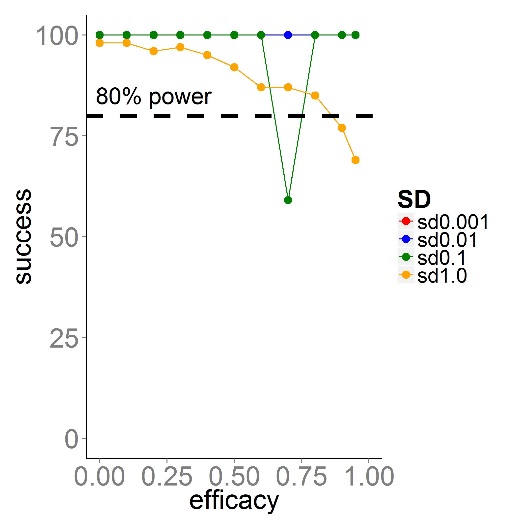

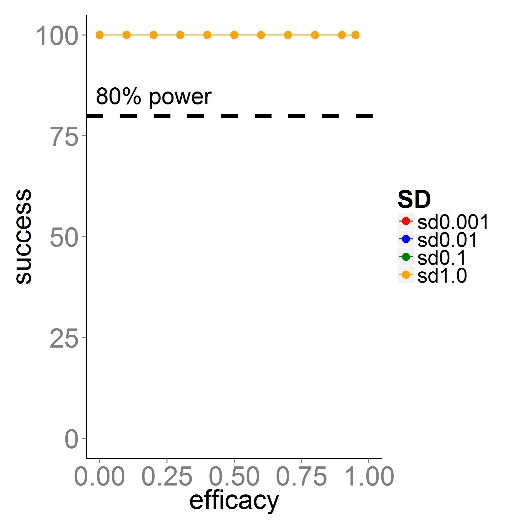

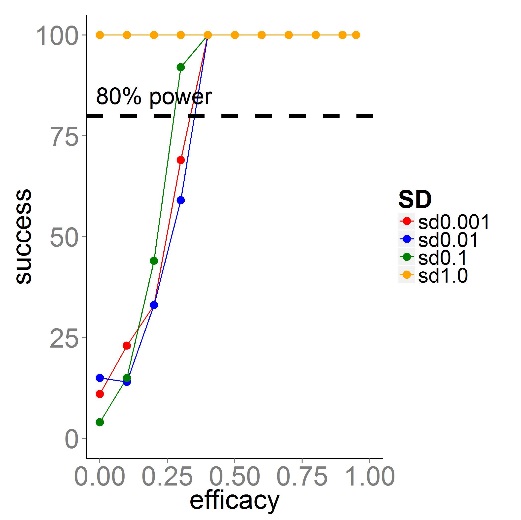

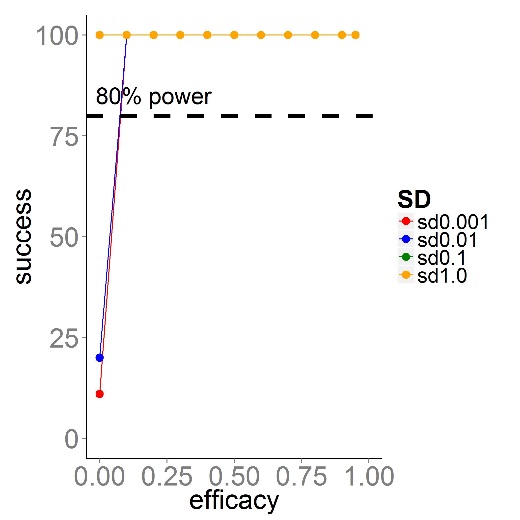


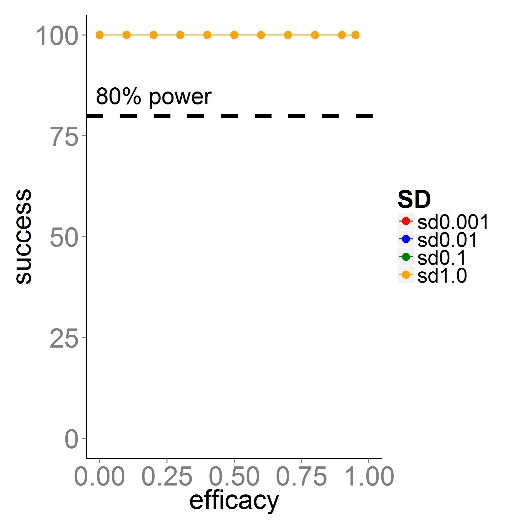

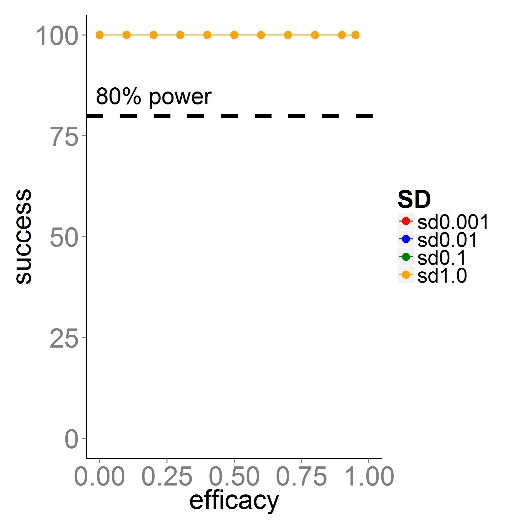

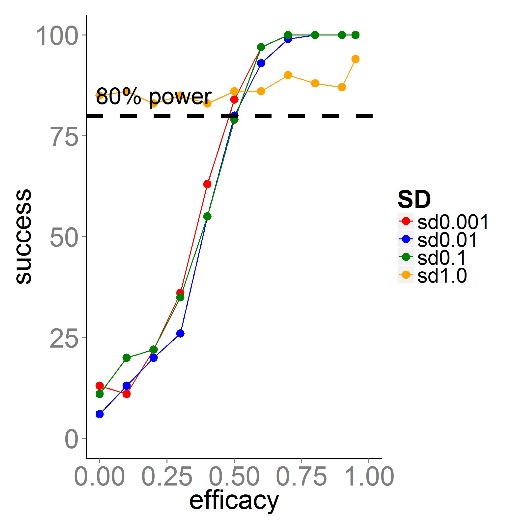

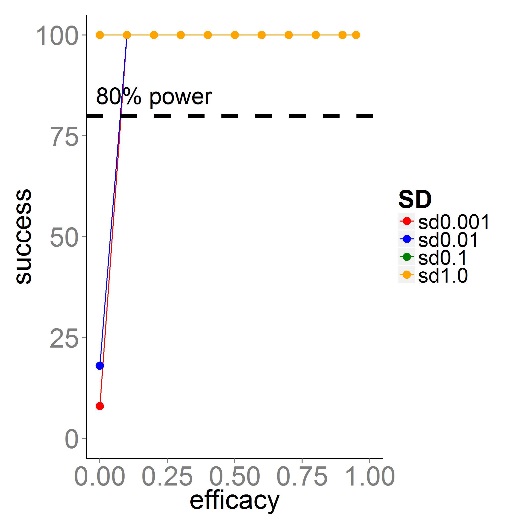


a

b

c

d

Fig C: Experiment 2: Individual variability in response to treatment. Treatment acts on virus clearance rate. Plots show the number of successful trials out of 100 runs (y-axis) over the assumed mean efficacy of treatment (x-axis). The probability of success corresponds to the power of the trial. All patients had exactly the same course of natural infection. The efficacy for each patient (response) was drawn from a normal distribution with standard deviations as shown in the plot legends. Upper row: trials with 50 patients. Lower row: trials with 100 patients. a, e: endpoint viral load area under the curve (AUC) from qPCR measurements. b, f: endpoint AUC from TCID_50_ measurements. c, g: endpoint AUC of simulated viral load. d, h: endpoint temperature AUC.

e

f

g

h


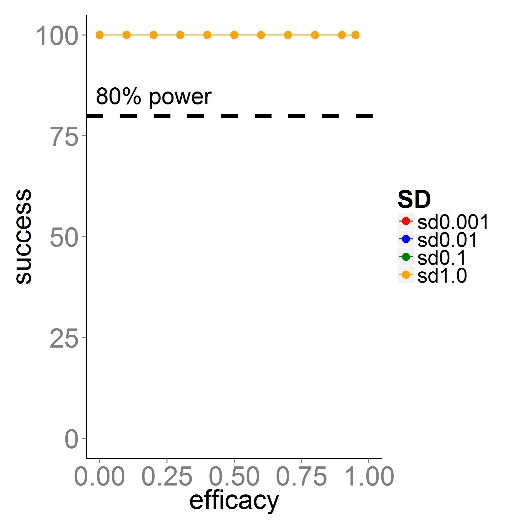

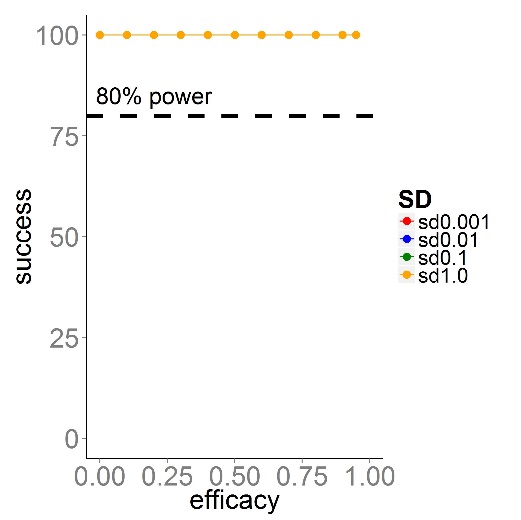

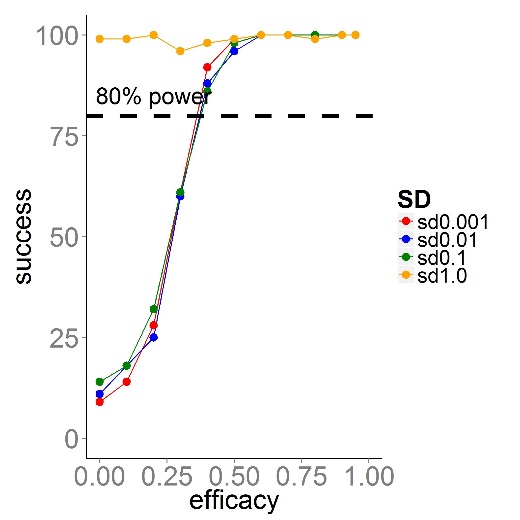

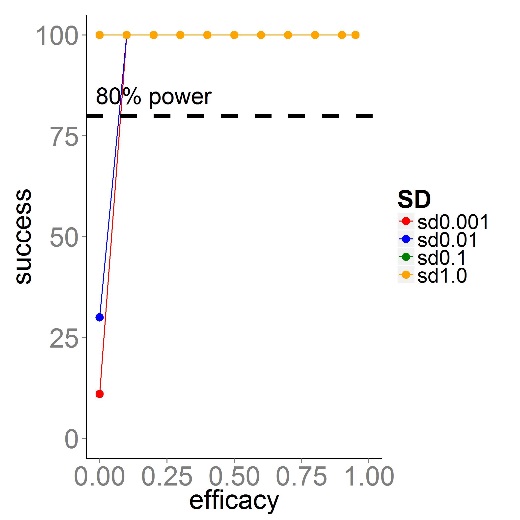


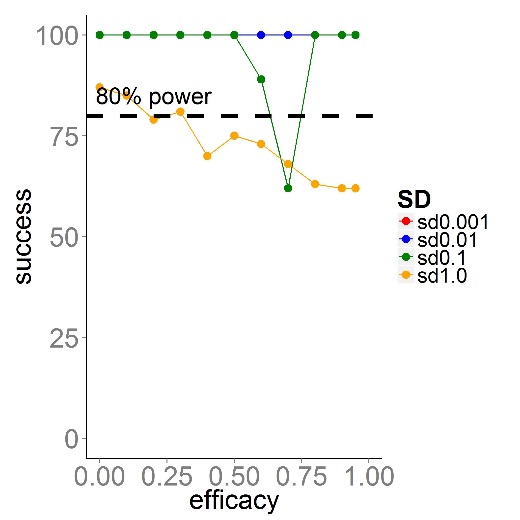

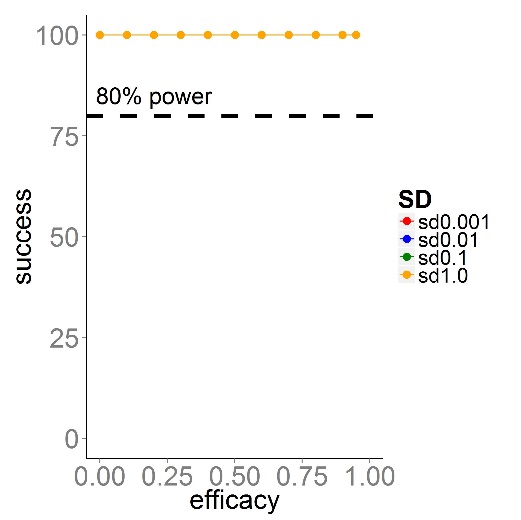

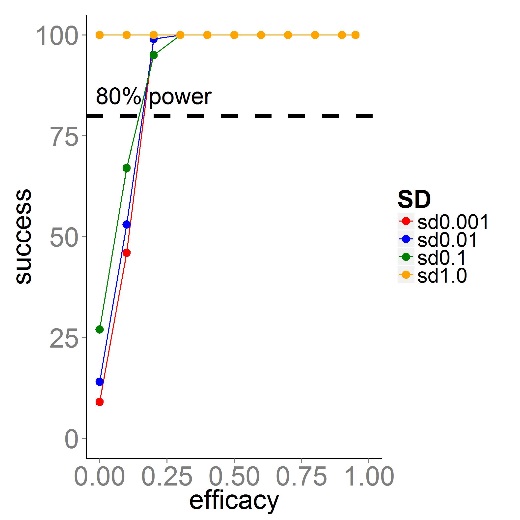

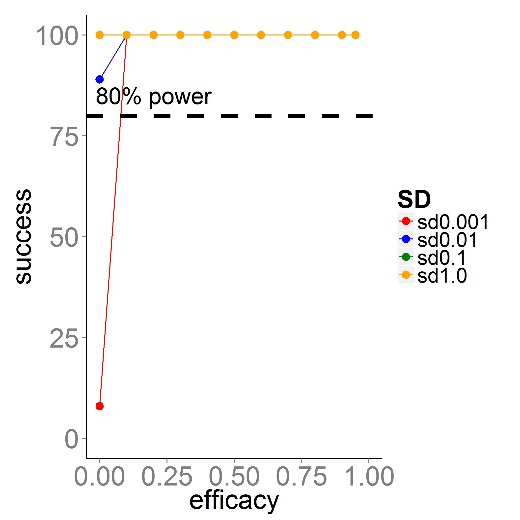


Fig D: Experiment 2: Individual variability in response to treatment. Treatment acts on virus production rate. Plots show the number of successful trials out of 100 runs (y-axis) over the assumed mean efficacy of treatment (x-axis). The probability of success corresponds to the power of the trial. All patients had exactly the same course of natural infection. The efficacy for each patient (response) was drawn from a normal distribution with standard deviations as shown in the plot legends. Upper row: trials with 50 patients. Lower row: trials with 100 patients. a, e: endpoint viral load area under the curve (AUC) from qPCR measurements. b, f: endpoint AUC from TCID_50_ measurements. c, g: endpoint AUC of simulated viral load. d, h: endpoint temperature AUC.

h

g

f

a

b

c

d

e


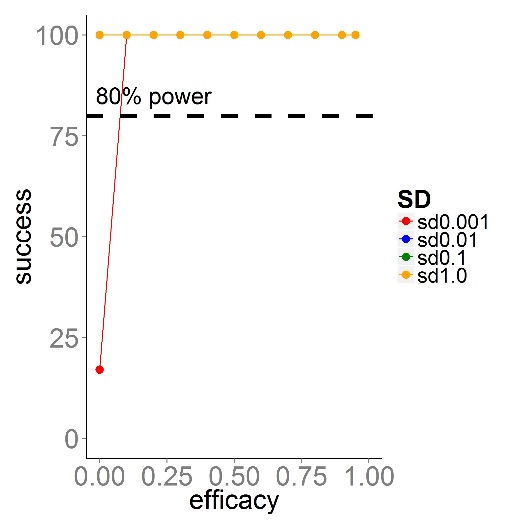


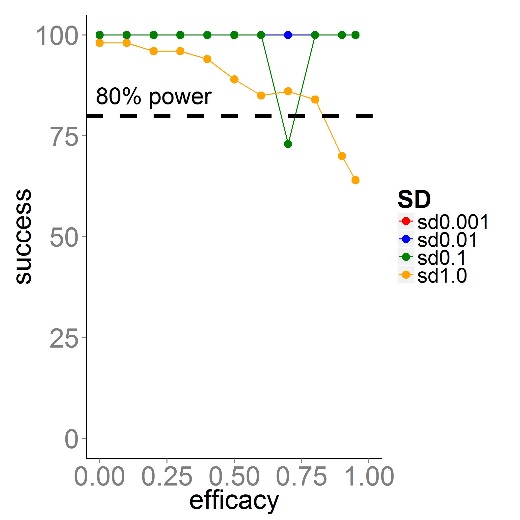

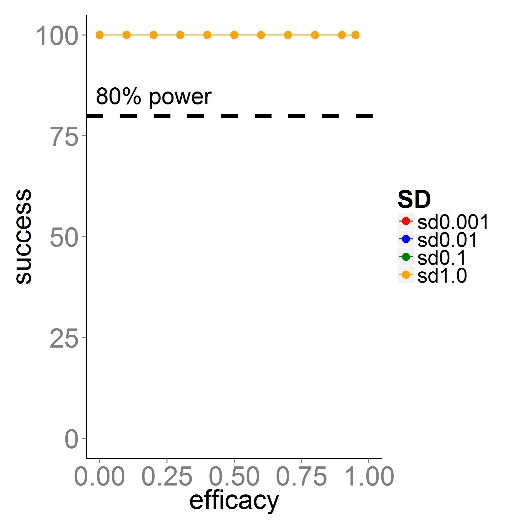

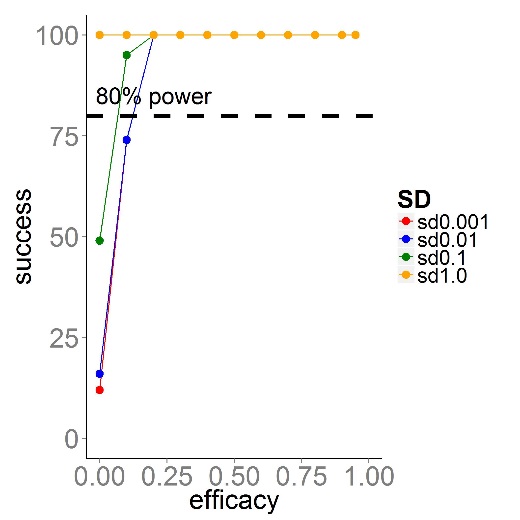


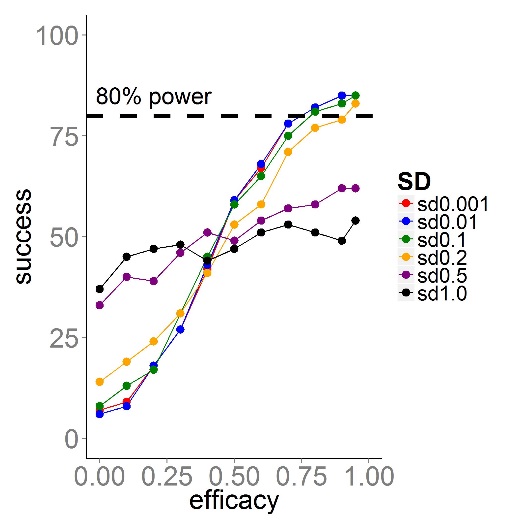

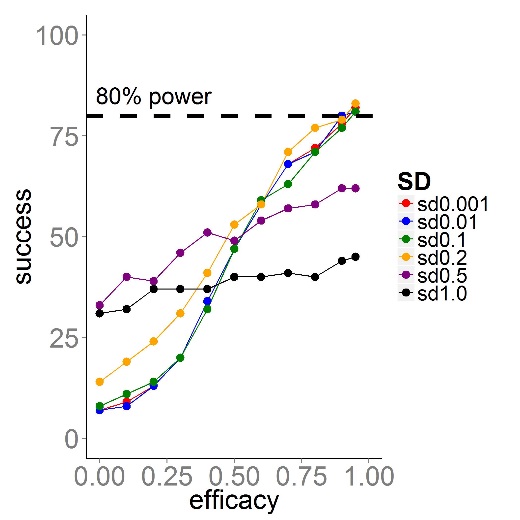

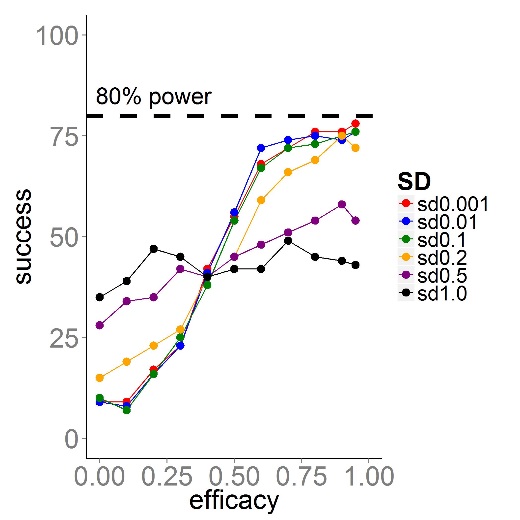

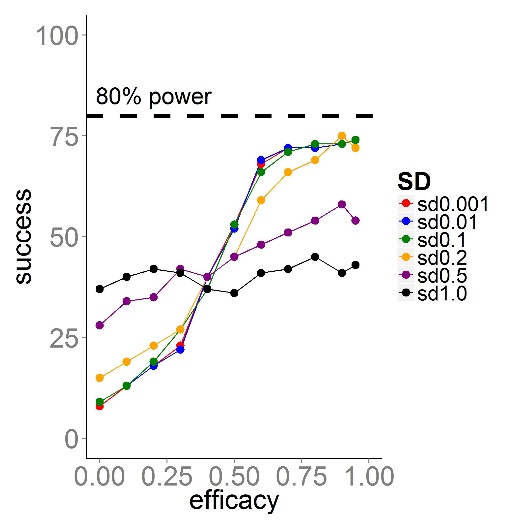


h

g

f

e

d

c

b

a

Fig E: Experiment 3: Individual variability in natural infection and response to treatment. Treatment acts on all model parameters. Plots show the number of successful trials out of 100 runs (y-axis) over the assumed mean efficacy of treatment (x-axis). The probability of success corresponds to the power of the trial. The parameters determining the course of natural infection were drawn from the same random number distributions for each patient as explained in the main text. The efficacy for each patient (response) was drawn from a normal distribution with standard deviations as shown in the plot legends. Upper row: trials with 50 patients. Lower row: trials with 100 patients. a, e: endpoint viral load area under the curve (AUC) from qPCR measurements. b, f: endpoint AUC from TCID_50_ measurements. c, g: endpoint AUC of simulated viral load. d, h: endpoint temperature AUC.


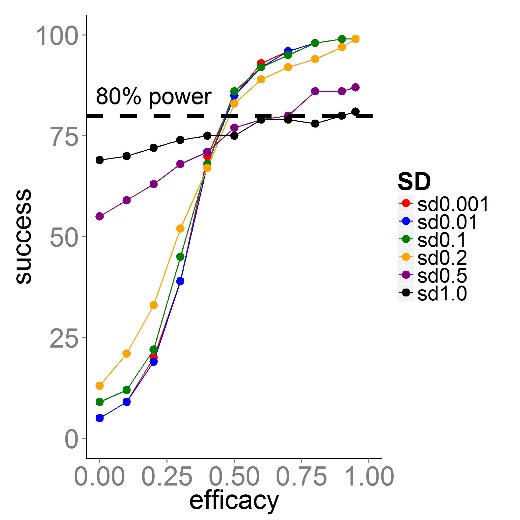

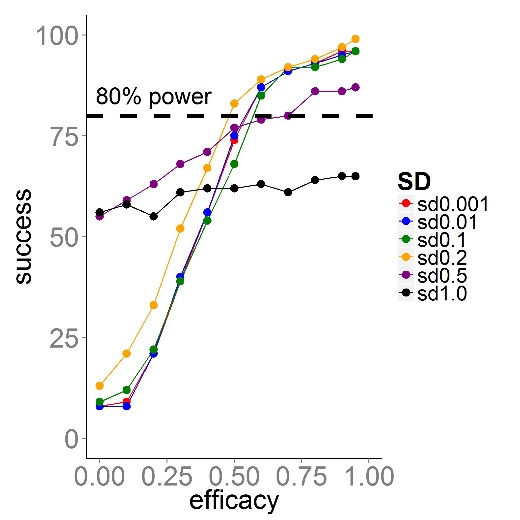

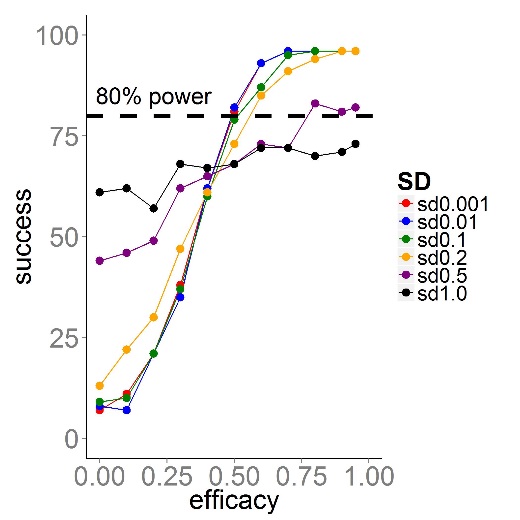

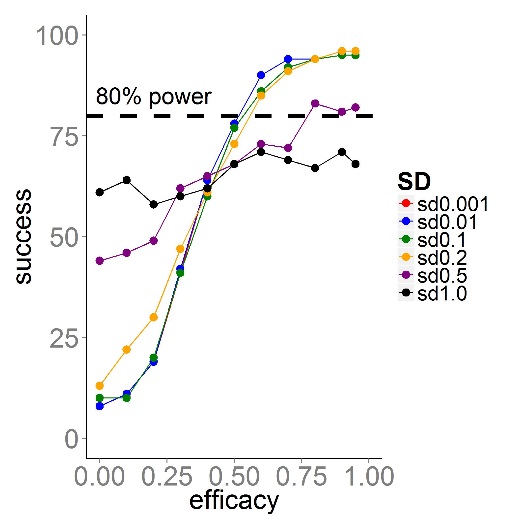


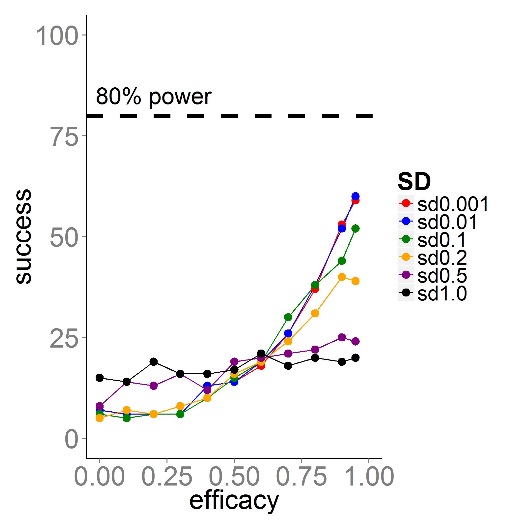

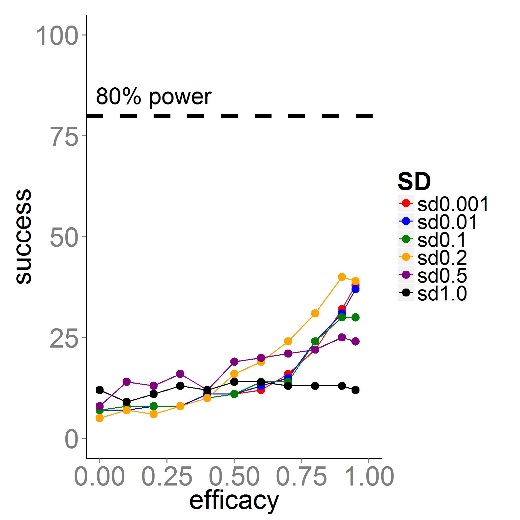

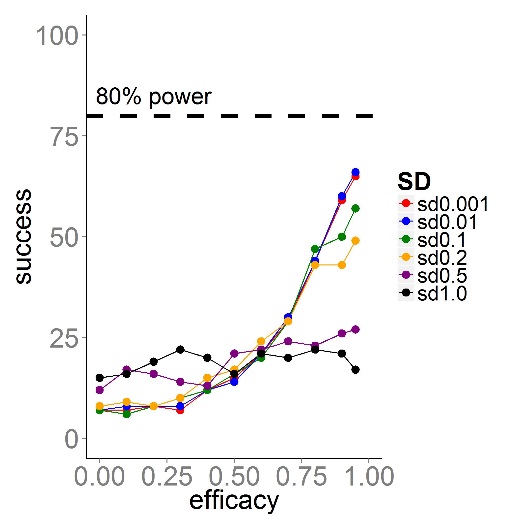

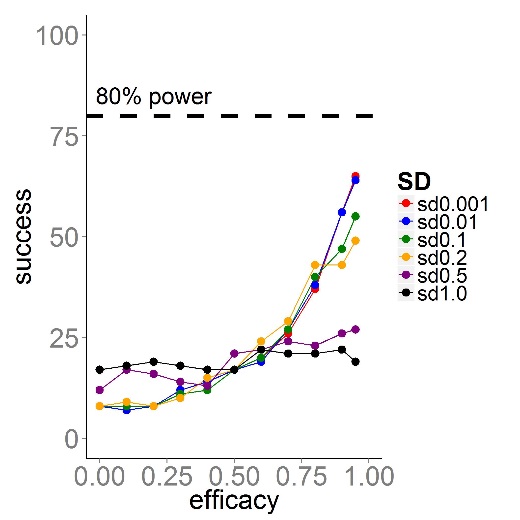


h

g

f

e

d

c

b

a

Fig F: Experiment 3: Individual variability in natural infection and response to treatment. Treatment acts on infection rate. Plots show the number of successful trials out of 100 runs (y-axis) over the assumed mean efficacy of treatment (x-axis). The probability of success corresponds to the power of the trial. The parameters determining the course of natural infection were drawn from the same random number distributions for each patient as explained in the main text. The efficacy for each patient (response) was drawn from a normal distribution with standard deviations as shown in the plot legends. Upper row: trials with 50 patients. Lower row: trials with 100 patients. a, e: endpoint viral load area under the curve (AUC) from qPCR measurements. b, f: endpoint AUC from TCID_50_ measurements. c, g: endpoint AUC of simulated viral load. d, h: endpoint temperature AUC.


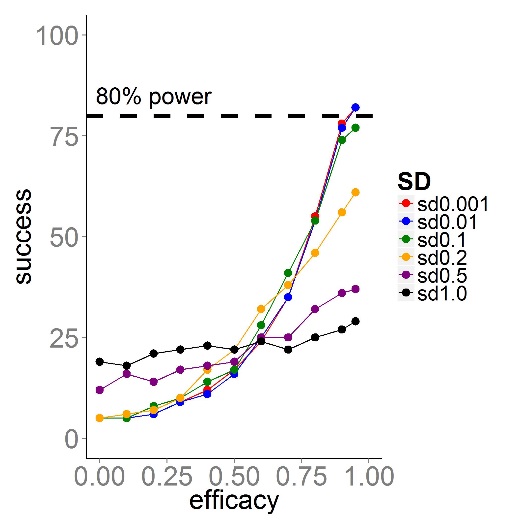

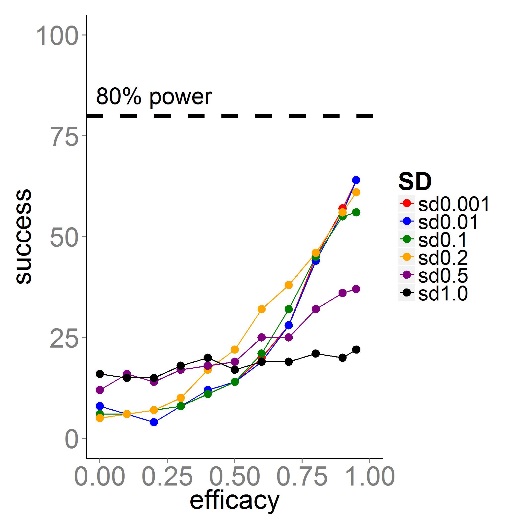

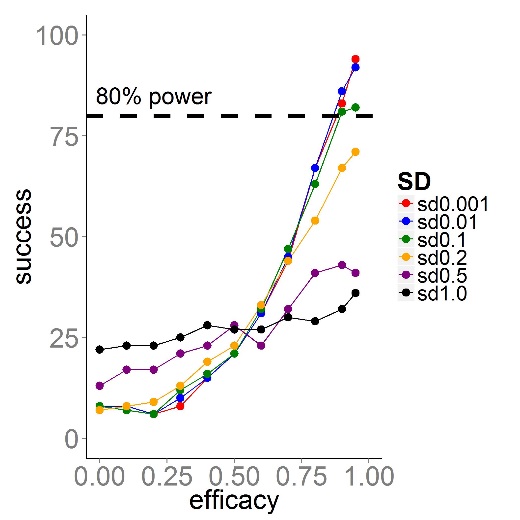

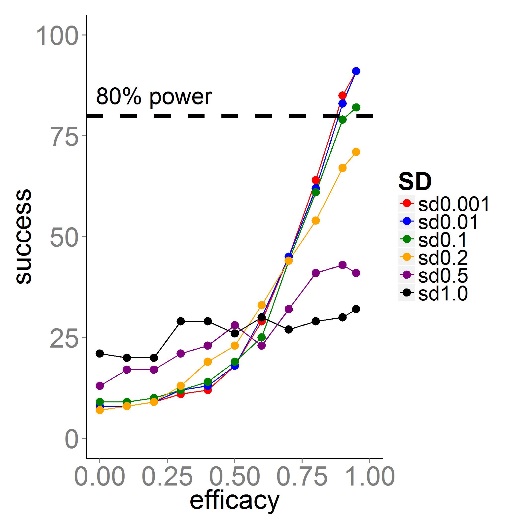


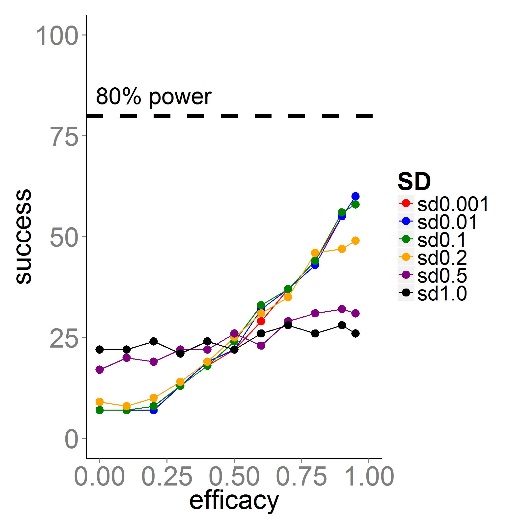

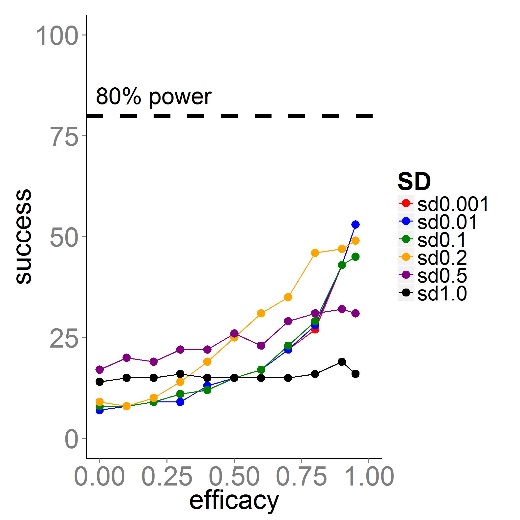

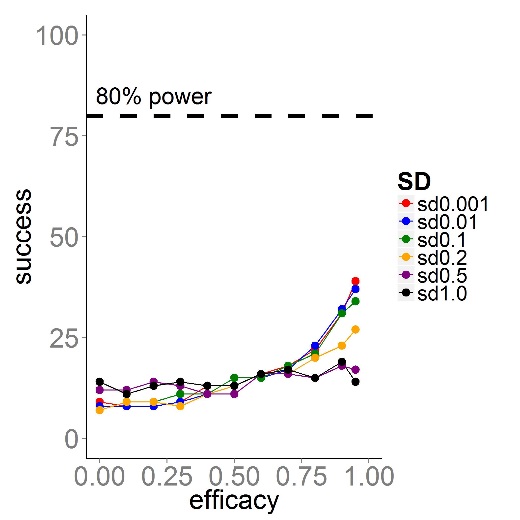

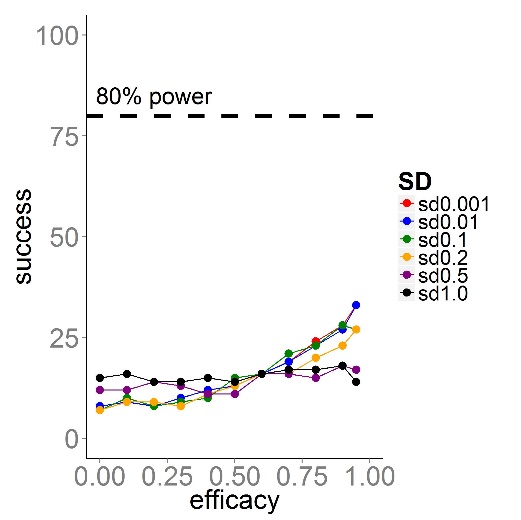


Fig G: Experiment 3: Individual variability in natural infection and response to treatment. Treatment acts on virus clearance rate. Plots show the number of successful trials out of 100 runs (y-axis) over the assumed mean efficacy of treatment (x-axis). The probability of success corresponds to the power of the trial. The parameters determining the course of natural infection were drawn from the same random number distributions for each patient as explained in the main text. The efficacy for each patient (response) was drawn from a normal distribution with standard deviations as shown in the plot legends. Upper row: trials with 50 patients. Lower row: trials with 100 patients. a, e: endpoint viral load area under the curve (AUC) from qPCR measurements. b, f: endpoint AUC from TCID_50_ measurements. c, g: endpoint AUC of simulated viral load. d, h: endpoint temperature AUC.

h

g

f

e

d

c

b

a


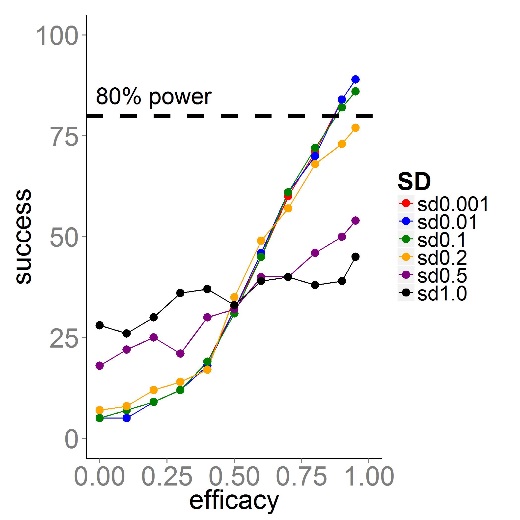

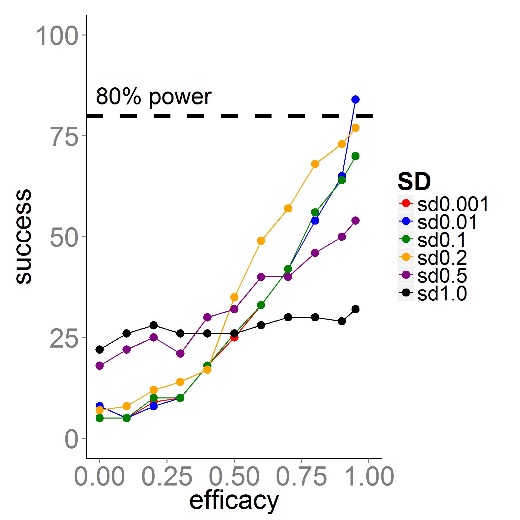

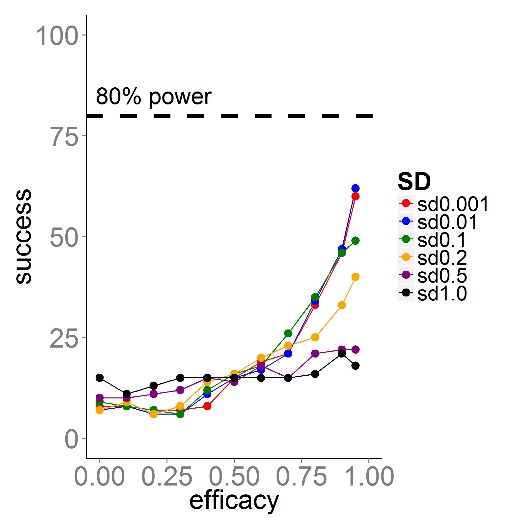

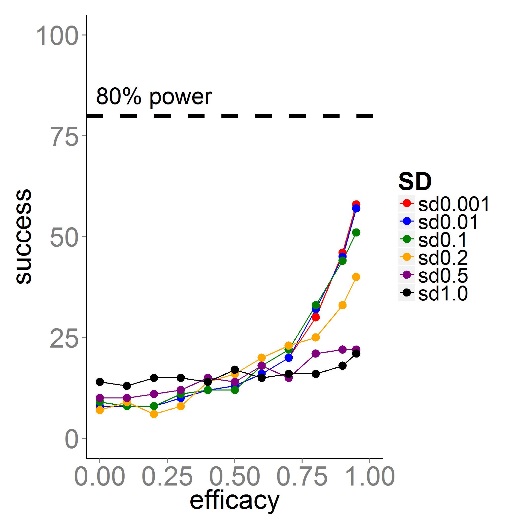


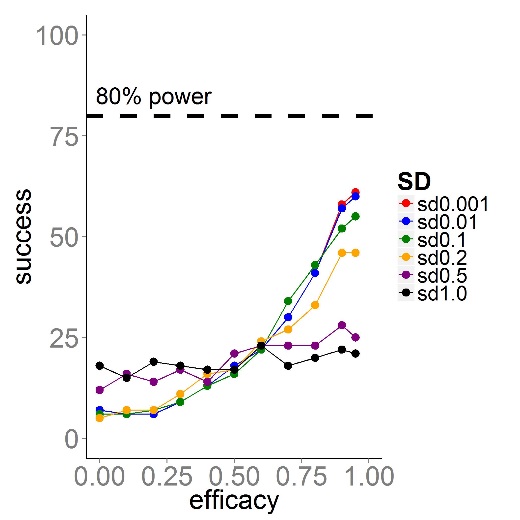

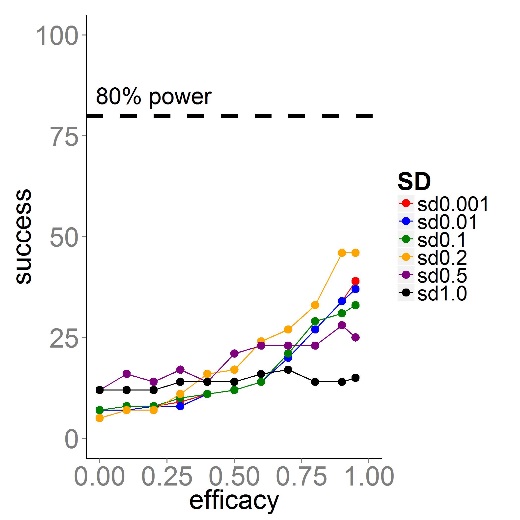

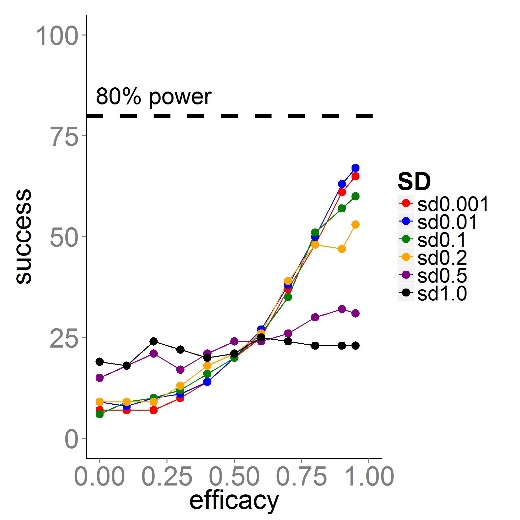

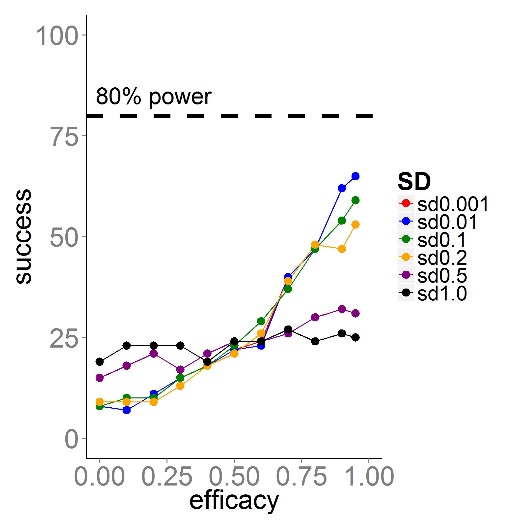


h

g

f

e

d

c

b

a

Fig H: Experiment 3: Individual variability in natural infection and response to treatment. Treatment acts on virus production rate. Plots show the number of successful trials out of 100 runs (y-axis) over the assumed mean efficacy of treatment (x-axis). The probability of success corresponds to the power of the trial. The parameters determining the course of natural infection were drawn from the same random number distributions for each patient as explained in the main text. The efficacy for each patient (response) was drawn from a normal distribution with standard deviations as shown in the plot legends. Upper row: trials with 50 patients. Lower row: trials with 100 patients. a, e: endpoint viral load area under the curve (AUC) from qPCR measurements. b, f: endpoint AUC from TCID_50_ measurements. c, g: endpoint AUC of simulated viral load. d, h: endpoint temperature AUC.


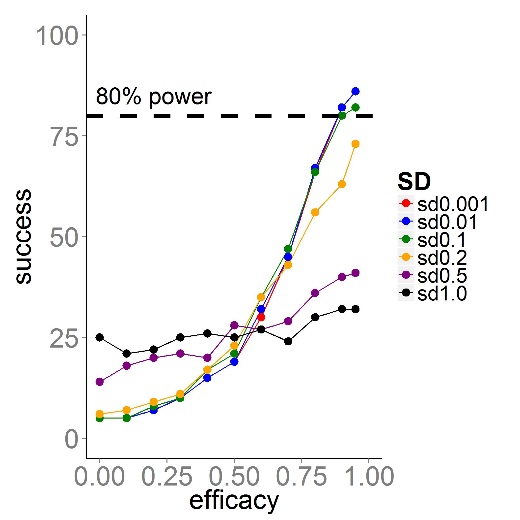

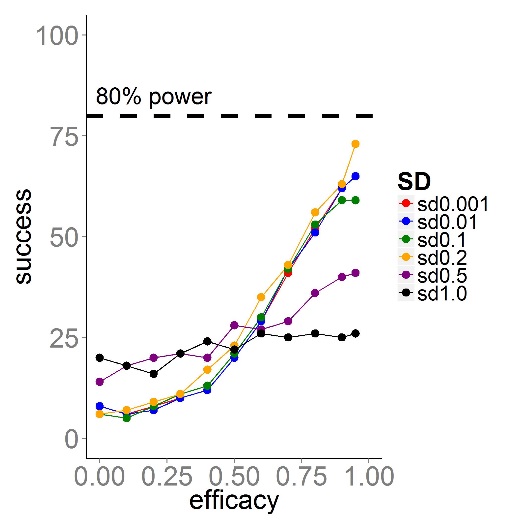

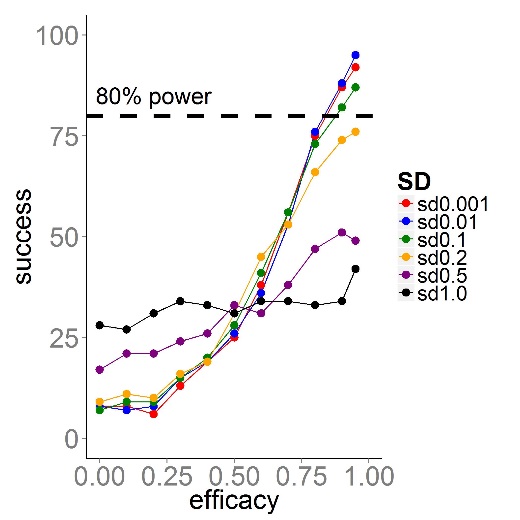

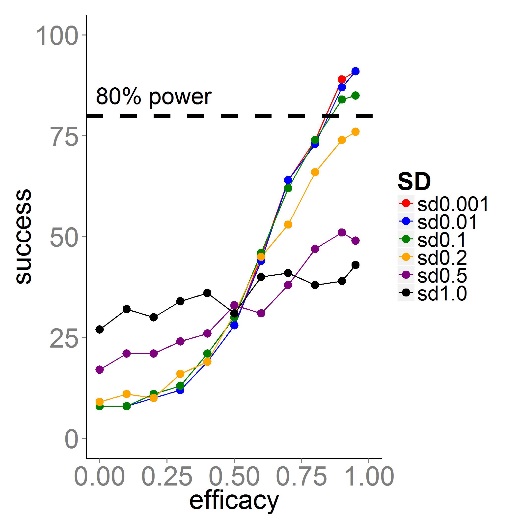


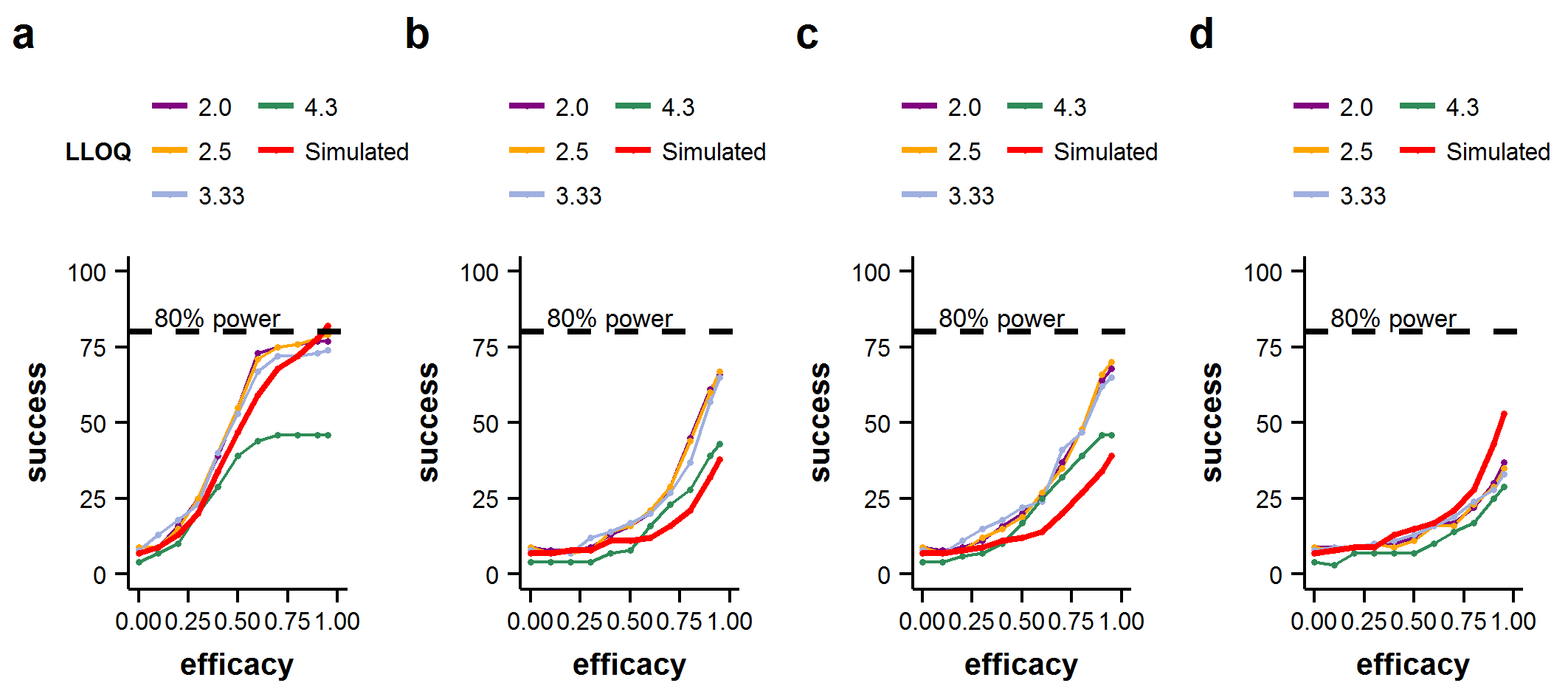


Fig I: Experiment 5: Sensitivity of viral load assay (qPCR). Plots show the number of successful trials out of 100 runs (y-axis) over the assumed mean efficacy of treatment (x-axis). The probability of success corresponds to the power of the trial. The parameters determining the course of natural infection were drawn from the same random number distributions for each patient as explained in the main text. The efficacy for each patient (response) was fixed to the same value for each patient in each run. Thin coloured lines show the power of the trial dependent on the efficacy of the treatment for qPCR viral load assays assuming different lower limits of quantification. The bold red line shows the power of the trial dependent on the efficacy of the treatment, if the simulated simulated viral load curve is considered. Very insensitive assays can greatly reduce the power of a trial, especially in potent drugs that act on several stages of the virus life cycle (a). Conversely, if the treatment acts on the infection rate (b) or the virus production rate (c), very sensitive viral load assays tend to give false positive results. Trials with 50 patients. a: treatment acts on all model parameters. b: treatment acts on the infection rate. c: treatment acts on the virus production rate. d: treatment acts on the virus clearance rate.


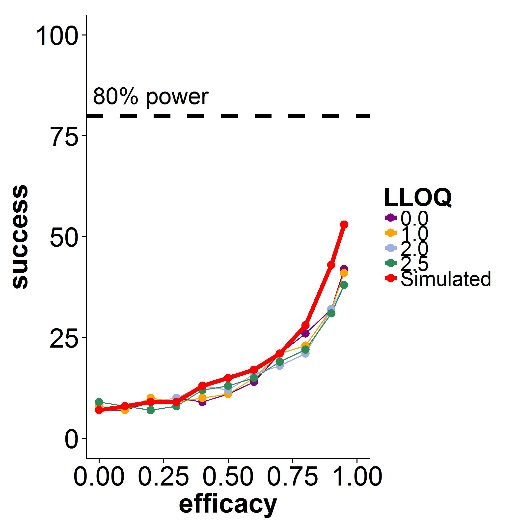

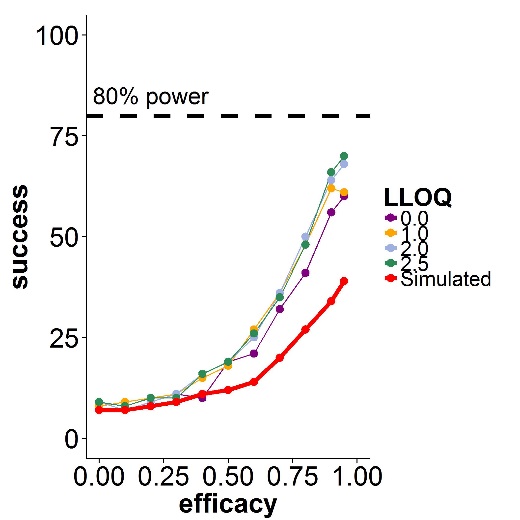

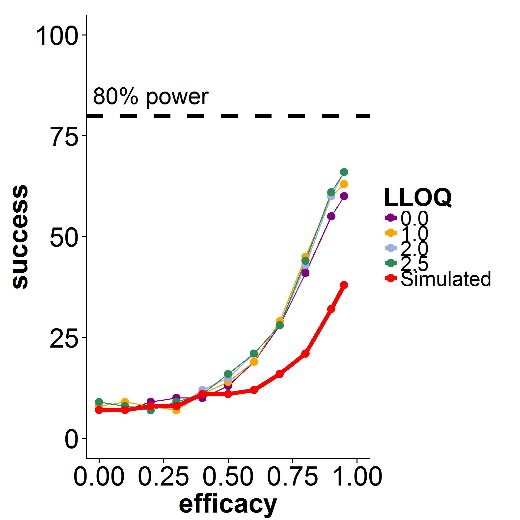

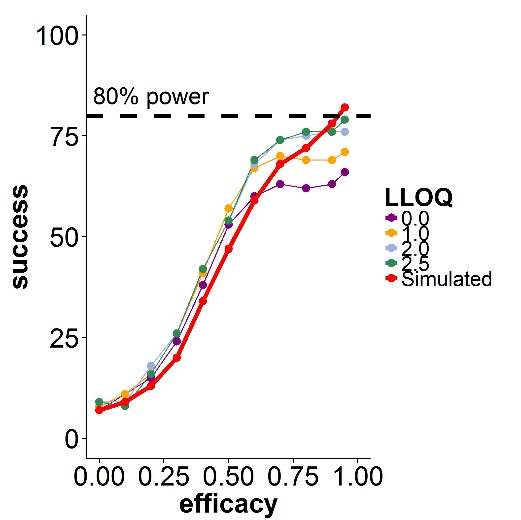


a

b

c

d

e

f

g

h


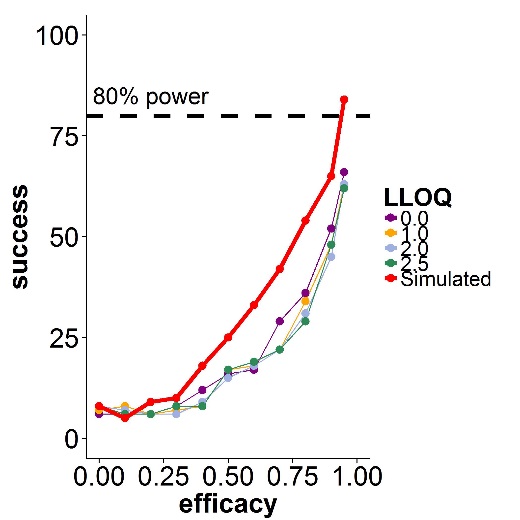

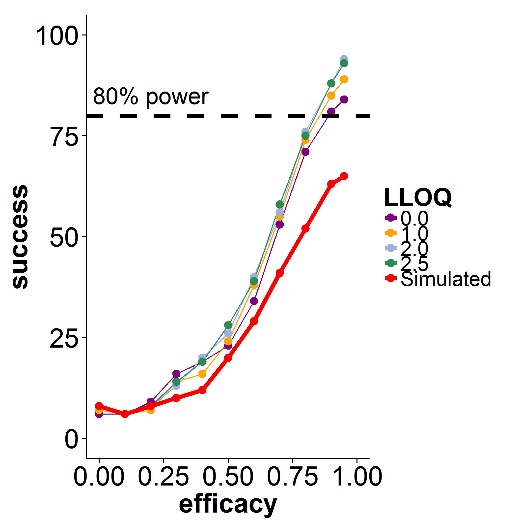

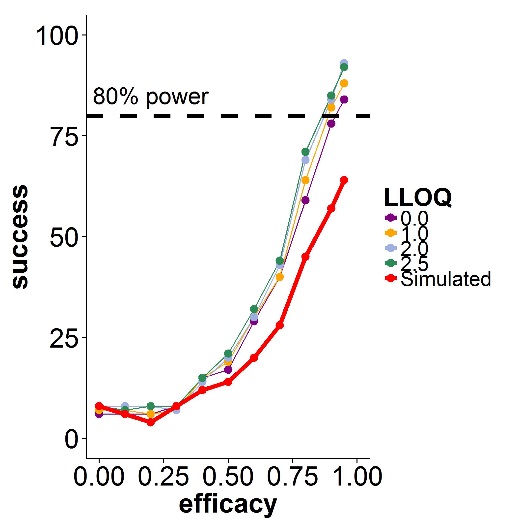

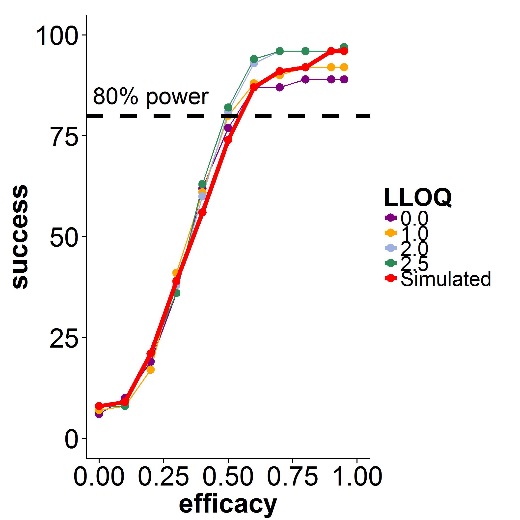


Fig J: Experiment 5: Sensitivity of viral load assay (TCID_50_). Plots show the number of successful trials out of 100 runs (y-axis) over the assumed mean efficacy of treatment (x-axis). The probability of success corresponds to the power of the trial. The parameters determining the course of natural infection were drawn from the same random number distributions for each patient as explained in the main text. The efficacy for each patient (response) was fixed to the same value for each patient in each run. Thin coloured lines show the power of the trial dependent on the efficacy of the treatment for TCID_50_ viral load assays assuming different lower limits of quantification. The bold red line shows the power of the trial dependent on the efficacy of the treatment if the simulated viral load curve is considered. Upper row: trials with 50 patients. Lower row: trials with 100 patients. a, e: Treatment acts on all model parameters. b, f: treatment acts on the infection rate. c g: treatment acts on the virus production rate. d, h: treatment acts on the virus clearance rate.


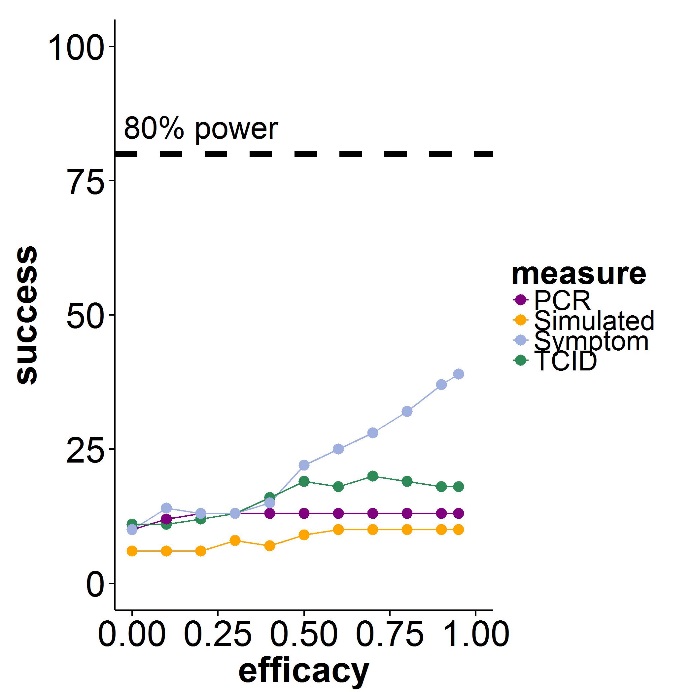

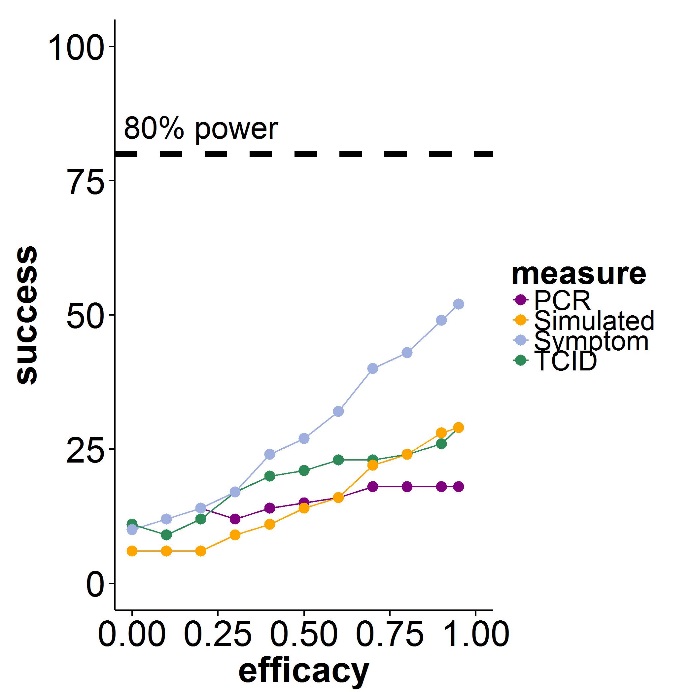

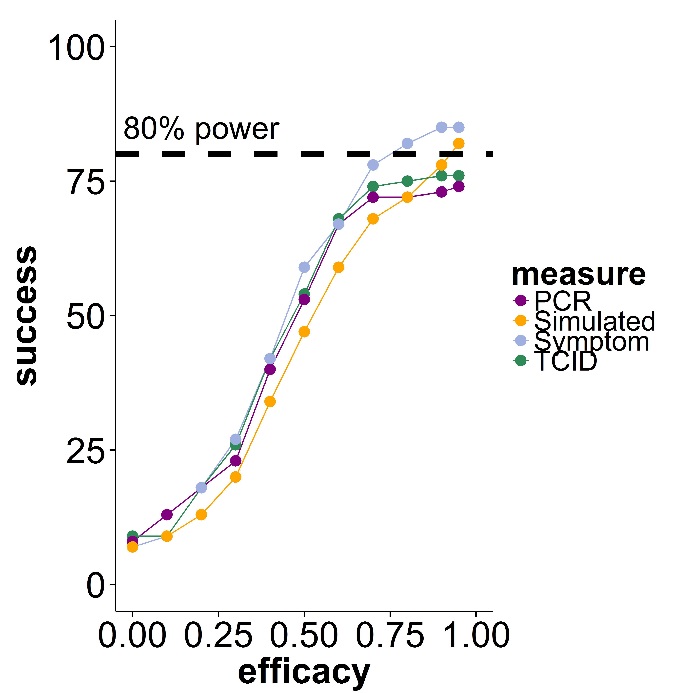


c

b

a

d

e

f

Fig K: Experiment 6: Day of Treatment. Treatment acts on all model parameters. Plots show the number of successful trials out of 100 runs (y-axis) over the assumed mean efficacy of treatment (x-axis). The probability of success corresponds to the power of the trial. The parameters determining the course of natural infection were drawn from the same random number distributions for each patient as explained in the main text. The efficacy for each patient (response) was fixed to the same value for each patient in each run. The later treatment is given, the lower the power of the trial. a, d: treatment on day 1; b, e: treatment on day 2; c, f: treatment on day 3; a, b, c: 50 patients in trial; d, e, f: 100 patients in trial.


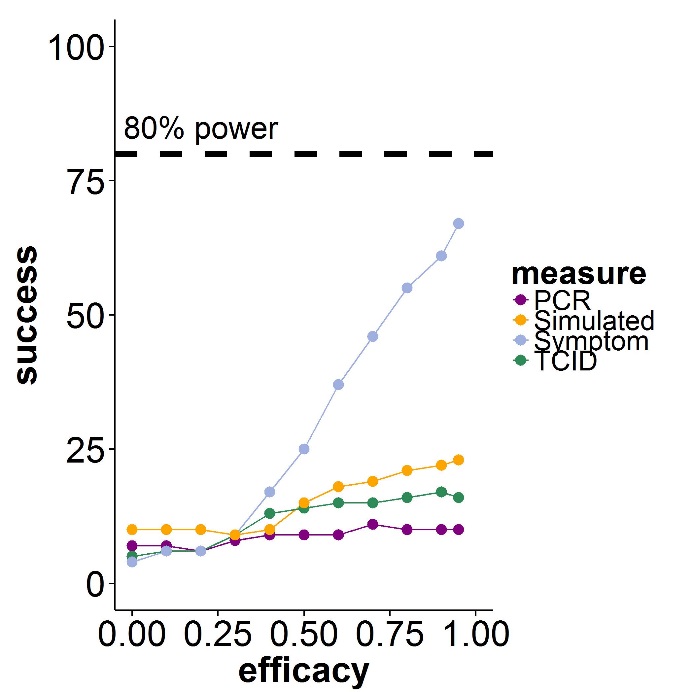

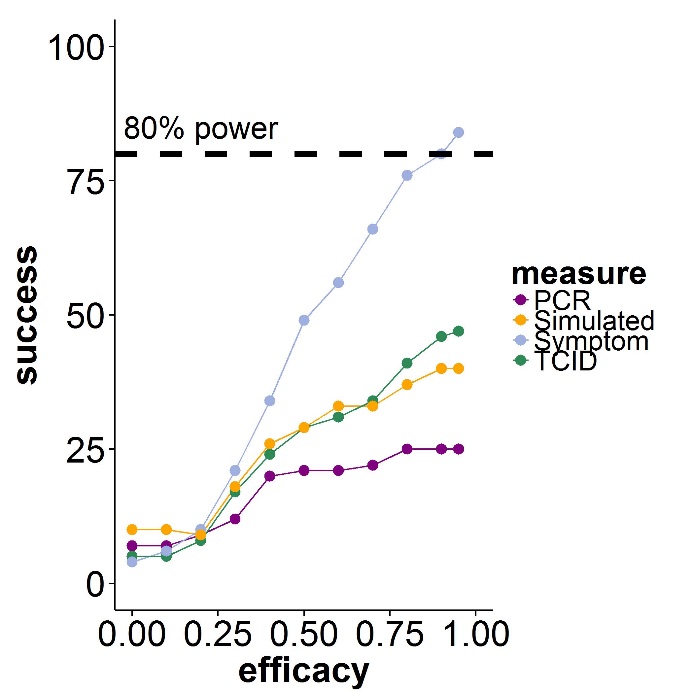

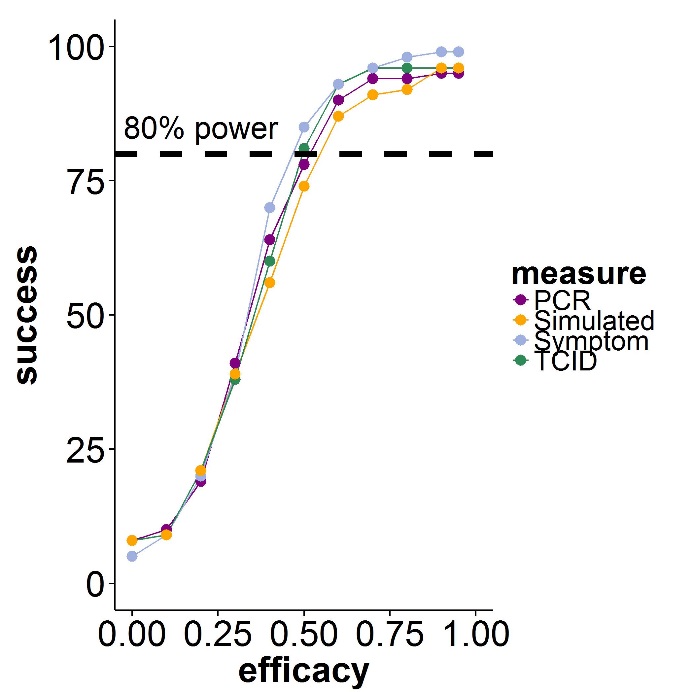


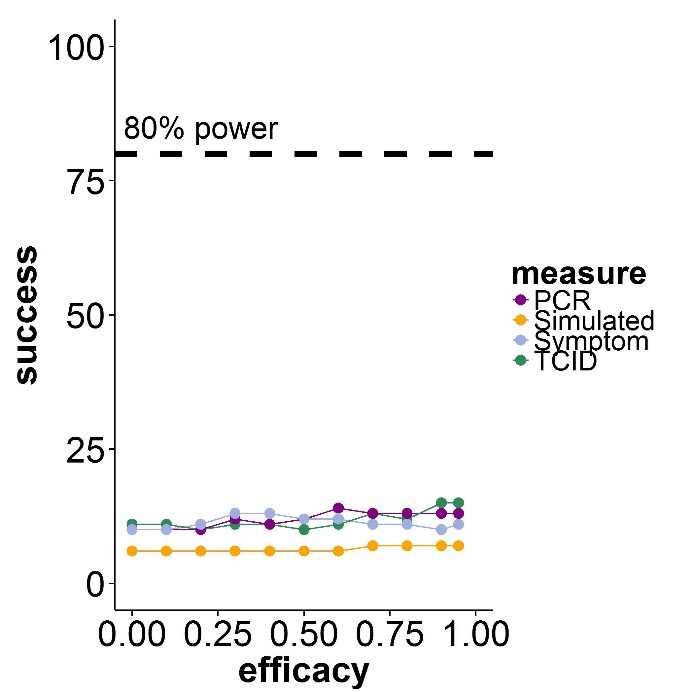

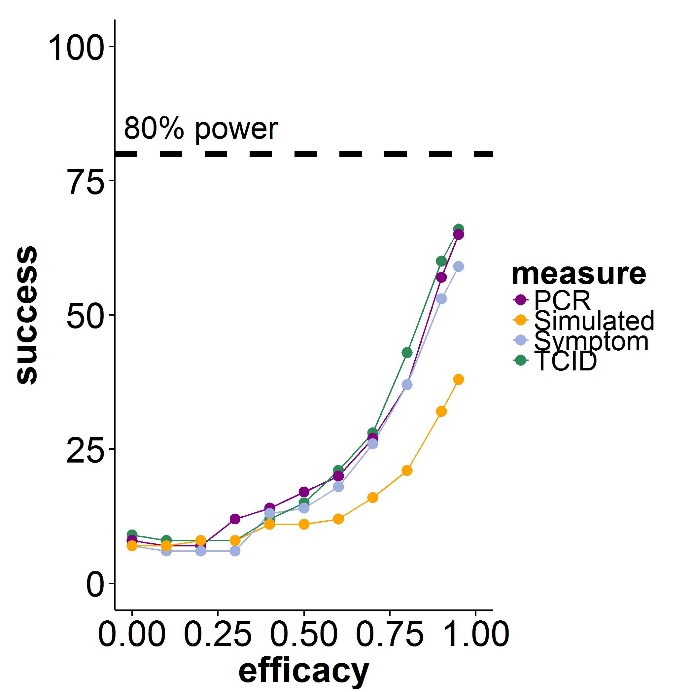

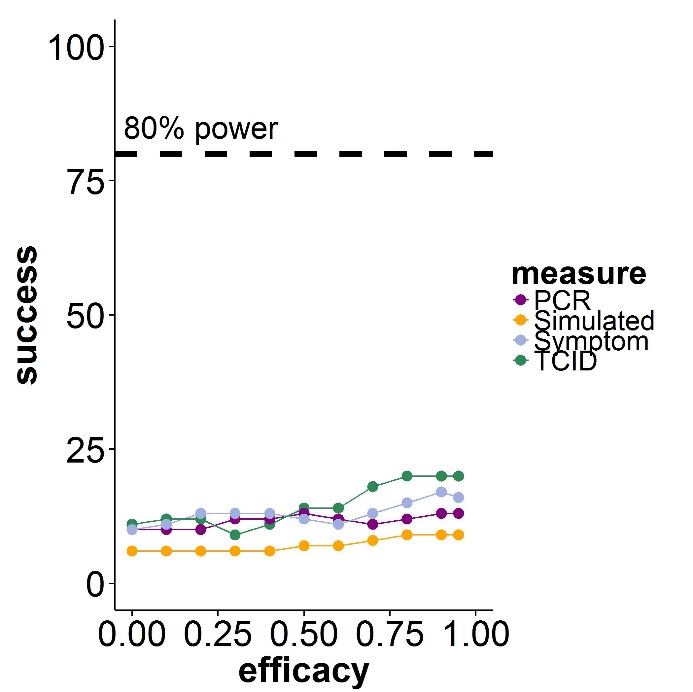


c

b

a

d

e

f

Fig L: Experiment 6: Day of Treatment. Treatment acts on infection rate. Plots show the number of successful trials out of 100 runs (y-axis) over the assumed mean efficacy of treatment (x-axis). The probability of success corresponds to the power of the trial. The parameters determining the course of natural infection were drawn from the same random number distributions for each patient as explained in the main text. The efficacy for each patient (response) was fixed to the same value for each patient in each run. The later treatment is given, the lower the power of the trial. a, d: treatment on day 1; b, e: treatment on day 2; c, f: treatment on day 3; a, b, c: 50 patients in trial; d, e, f: 100 patients in trial.


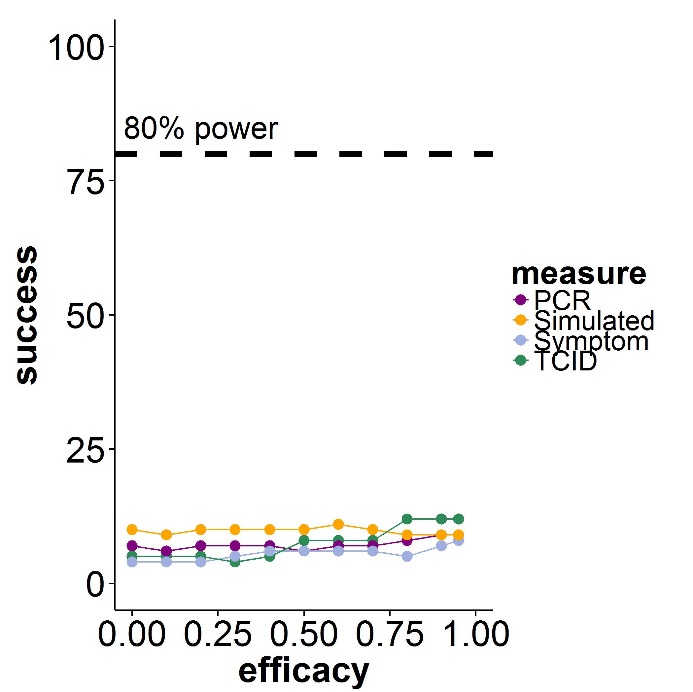

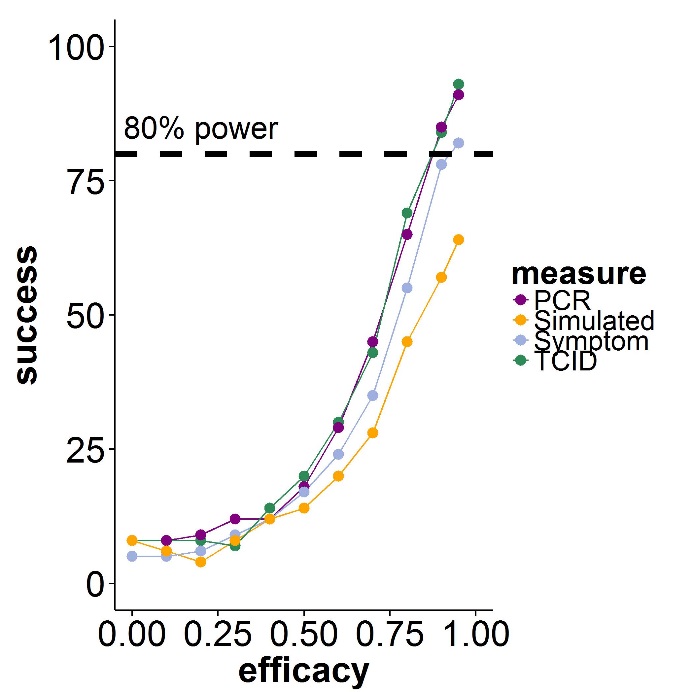

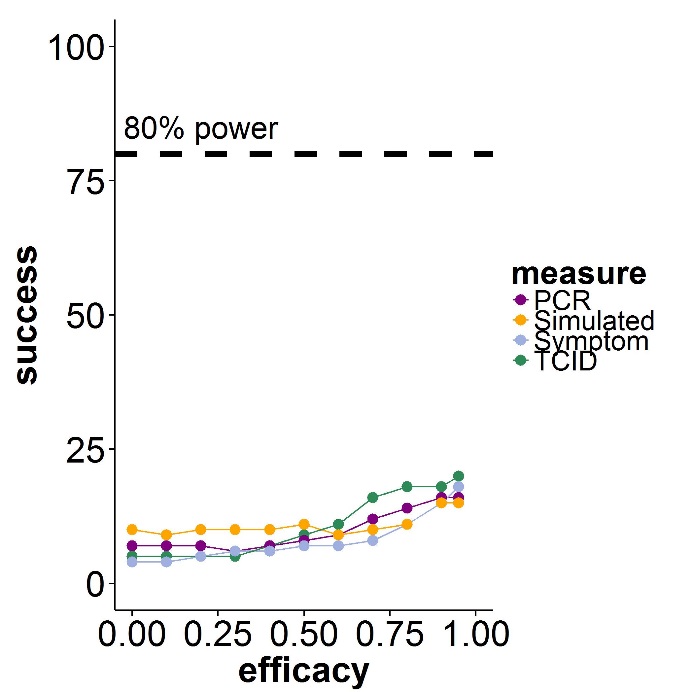


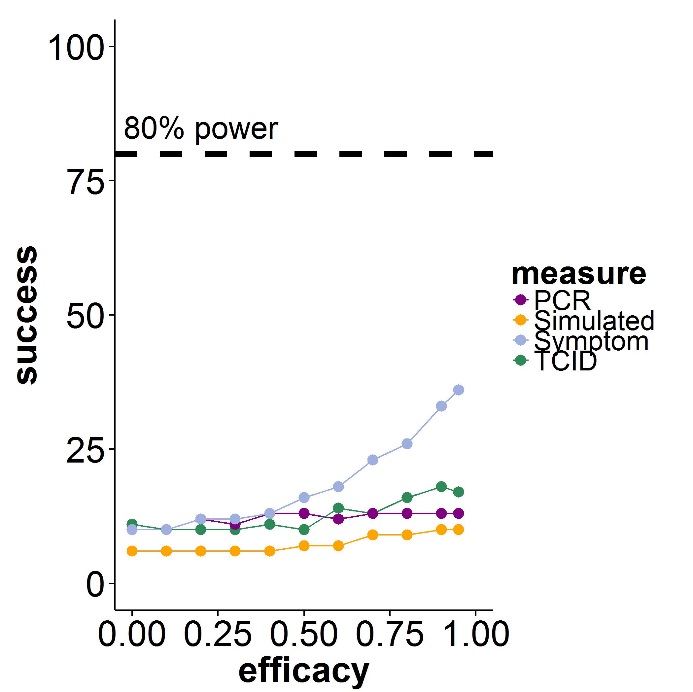

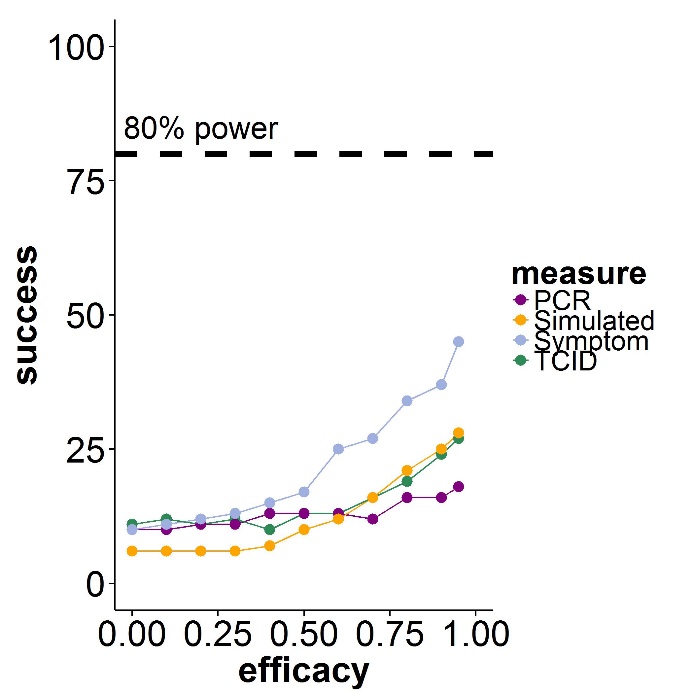

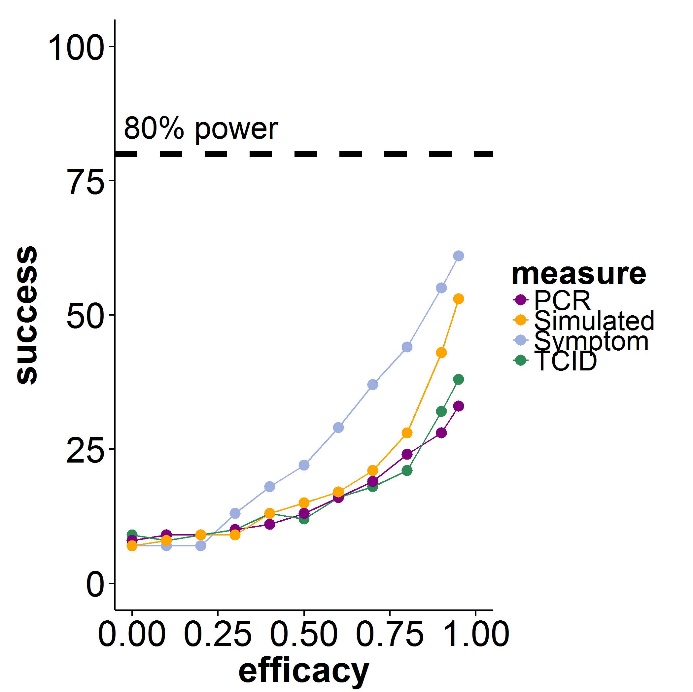


c

b

a

f

e

d

Fig M: Experiment 6: Day of Treatment. Treatment acts on virus clearance rate. Plots show the number of successful trials out of 100 runs (y-axis) over the assumed mean efficacy of treatment (x-axis). The probability of success corresponds to the power of the trial. The parameters determining the course of natural infection were drawn from the same random number distributions for each patient as explained in the main text. The efficacy for each patient (response) was fixed to the same value for each patient in each run. The later treatment is given, the lower the power of the trial. a, d: treatment on day 1; b, e: treatment on day 2; c, f: treatment on day 3; a, b, c: 50 patients in trial; d, e, f: 100 patients in trial.


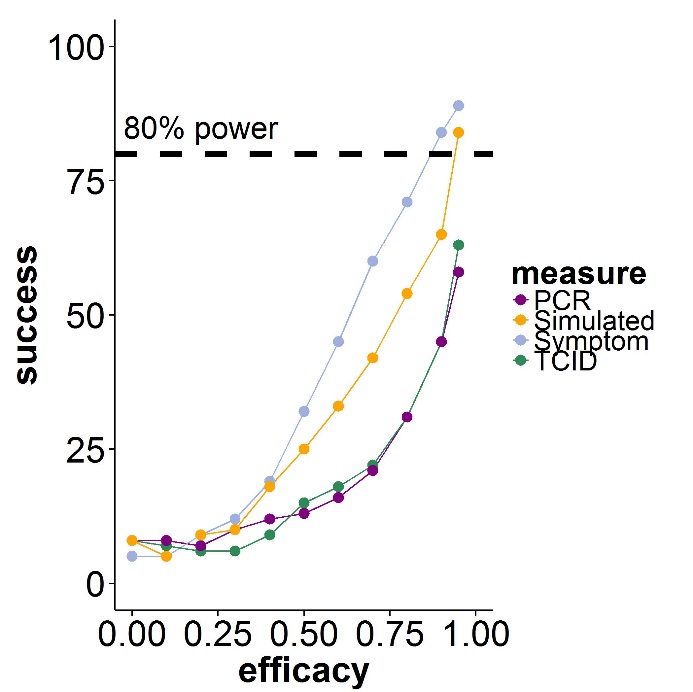


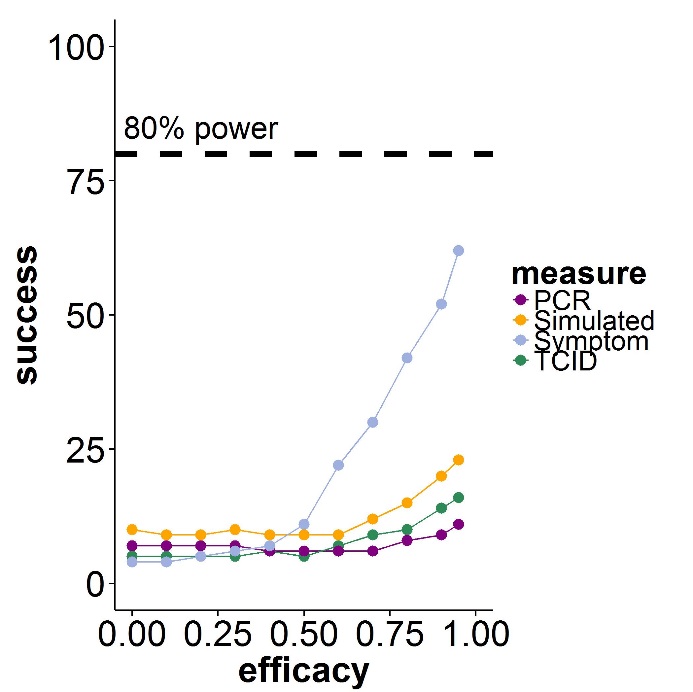

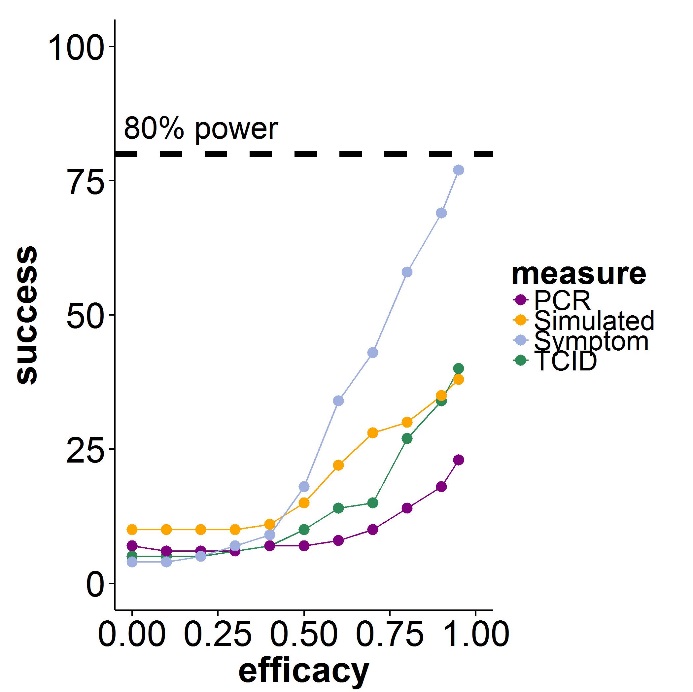


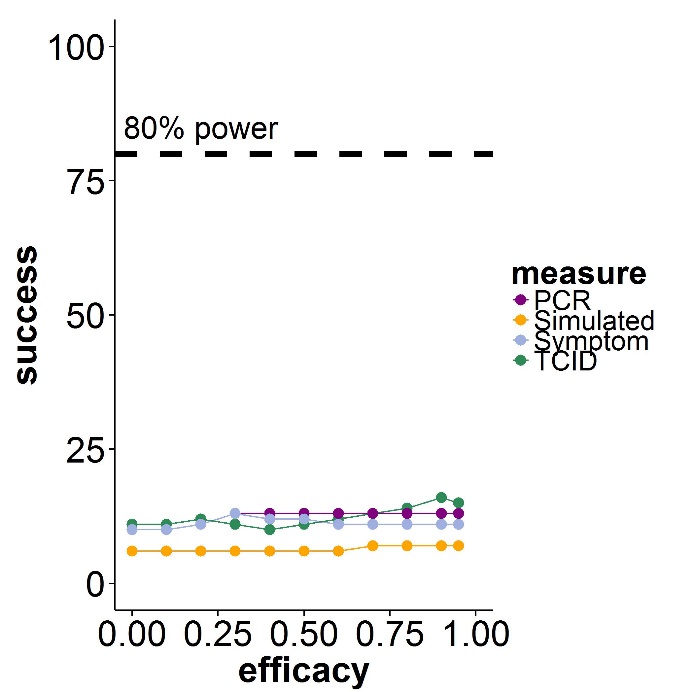

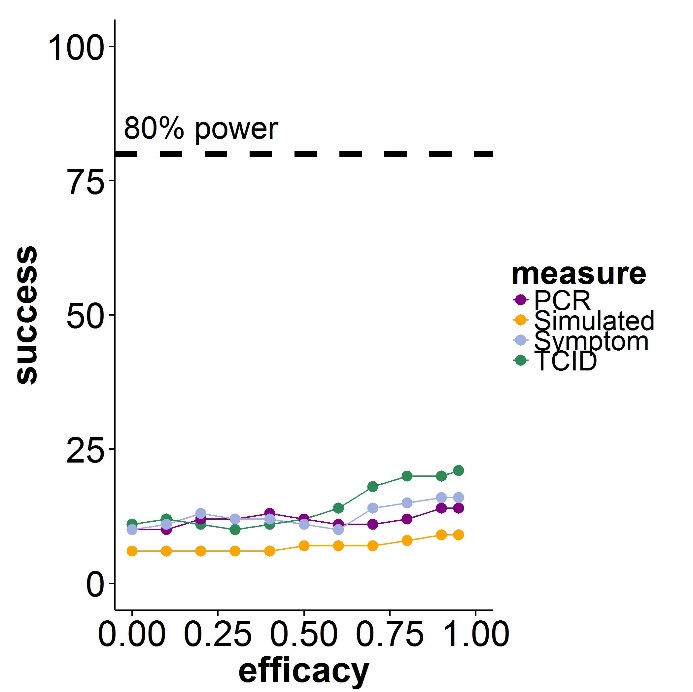

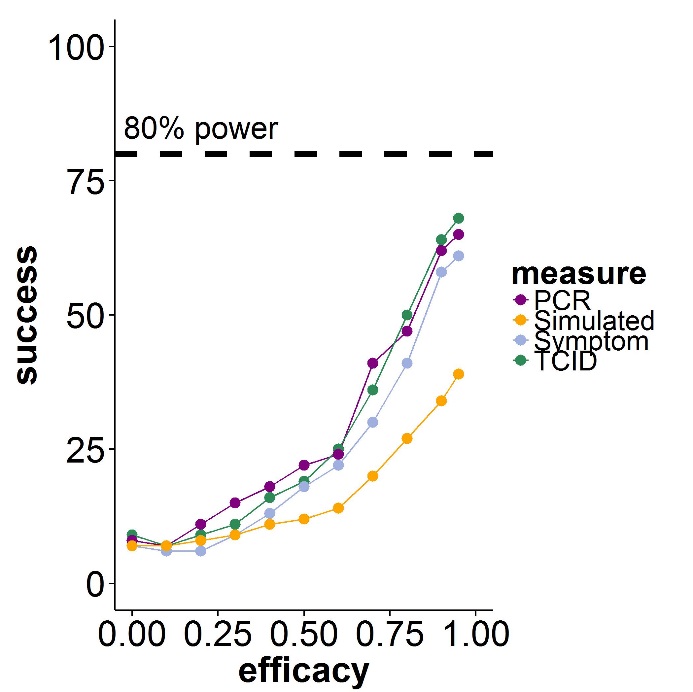


c

b

a

f

e

d

Fig N: Experiment 6: Day of Treatment. Treatment acts on virus production rate. Plots show the number of successful trials out of 100 runs (y-axis) over the assumed mean efficacy of treatment (x-axis). The probability of success corresponds to the power of the trial. The parameters determining the course of natural infection were drawn from the same random number distributions for each patient as explained in the main text. The efficacy for each patient (response) was fixed to the same value for each patient in each run. The later treatment is given, the lower the power of the trial. a, d: treatment on day 1; b, e: treatment on day 2; c, f: treatment on day 3; a, b, c: 50 patients in trial; d, e, f: 100 patients in trial.


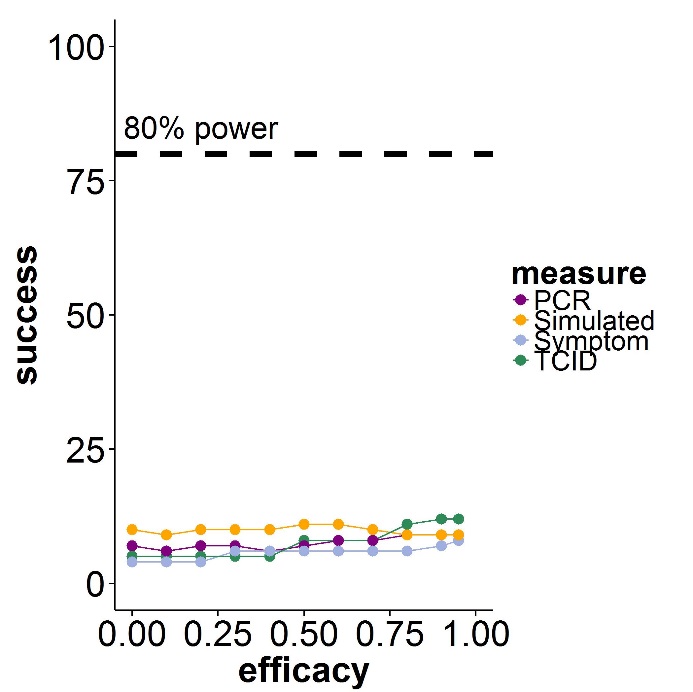

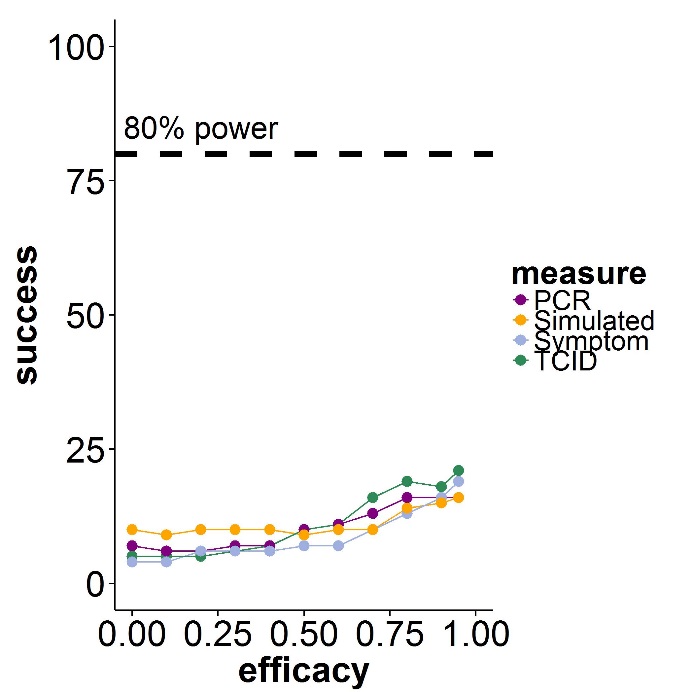

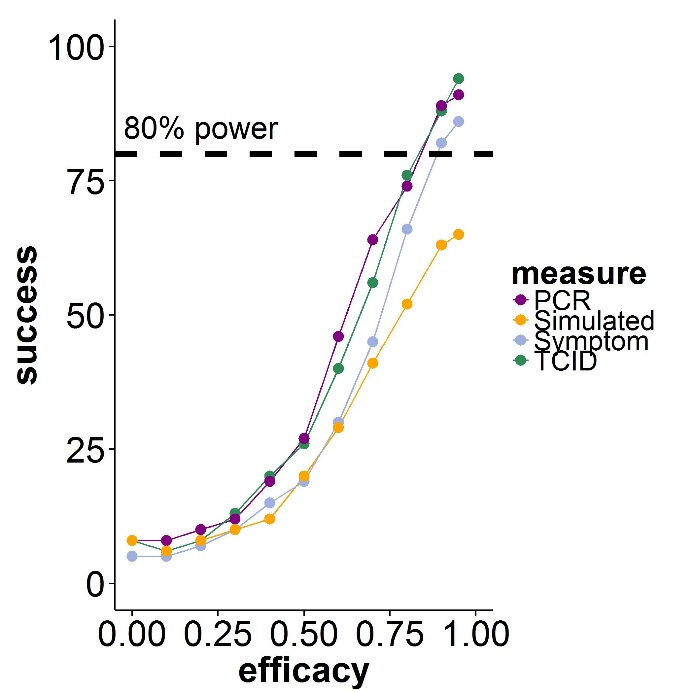


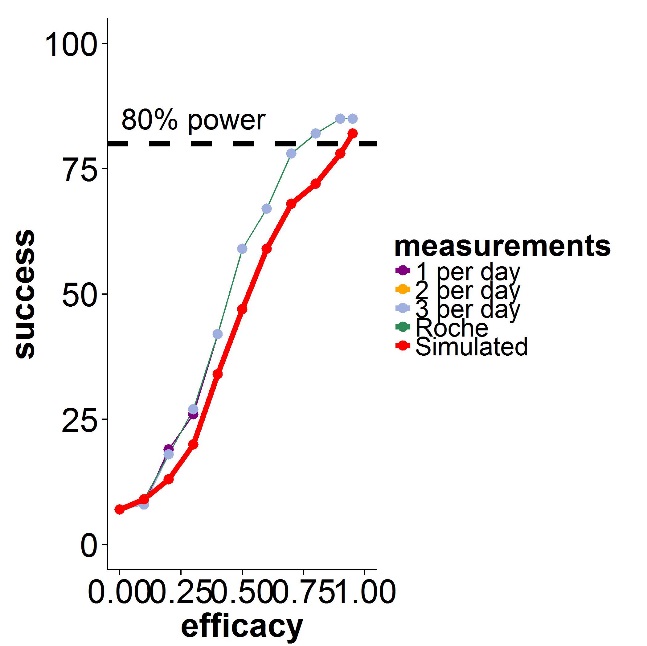

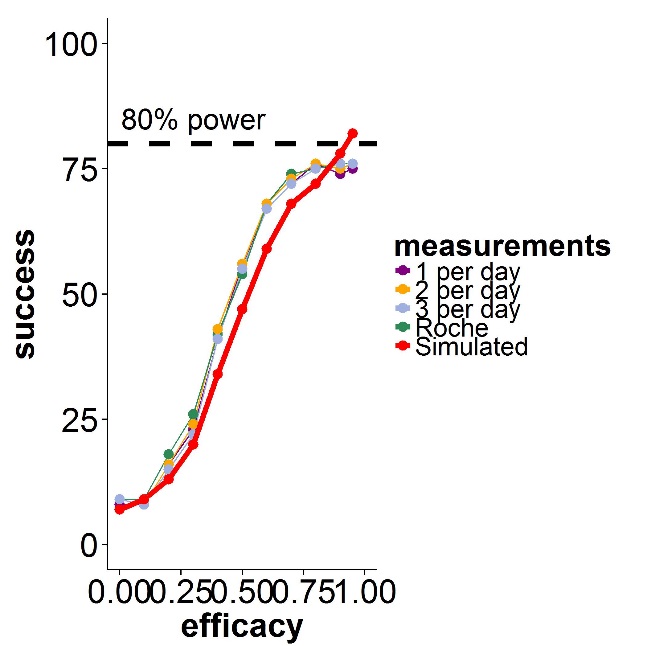

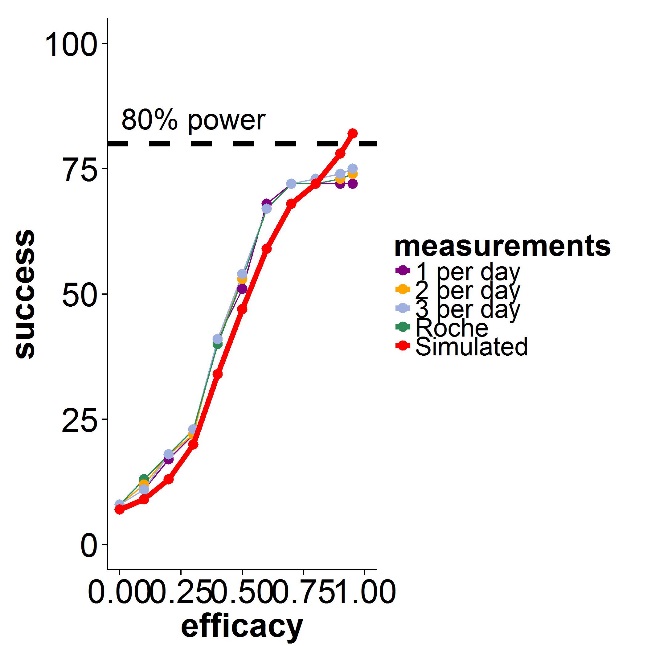


c

b

a

d

e

f

Fig O: Experiment 7: Frequency of endpoint measurements. Treatment acts on all model parameters. Plots show the number of successful trials out of 100 runs (y-axis) over the assumed mean efficacy of treatment (x-axis). The probability of success corresponds to the power of the trial. The parameters determining the course of natural infection were drawn from the same random number distributions for each patient as explained in the main text. The efficacy for each patient (response) was fixed to the same value for each patient in each run. Thin coloured lines show the power of trial dependent on the efficacy of the treatment for different measurement protocols. “Roche” means the measurement protocol as described in the Methods section of the main text. The bold red line shows the power of the trial depending on the efficacy of the treatment when the simulated viral load curve is considered. Taking more than one viral load measurement per day does not increase the power of the trial. The same applies to temperature (symptom measurements). a, d endpoint viral load area under the curve (AUC) from qPCR measurements; b, e: endpoint AUC from TCID_50_ measurements; c, f: endpoint temperature AUC; a, b, c: 50 patients in trial; d, e, f: 100 patients in trial.

a

b

c

d

e

f

Fig P: Experiment 7: Frequency of endpoint measurements. Treatment acts on the infection rate. Plots show the number of successful trials out of 100 runs (y-axis) over the assumed mean efficacy of treatment (x-axis). The probability of success corresponds to the power of the trial. The parameters determining the course of natural infection were drawn from the same random number distributions for each patient as explained in the main text. The efficacy for each patient (response) was fixed to the same value for each patient in each run. Thin coloured lines show the power of trial dependent on the efficacy of the treatment for different measurement protocols. “Roche” means the measurement protocol as described in the Methods section of the main text. The bold red line shows the power of the trial depending on the efficacy of the treatment when the simulated viral load curve is considered. Taking more than one viral load measurement per day does not increase the power of the trial. The same applies to temperature (symptom measurements a, d endpoint viral load area under the curve (AUC) from qPCR measurements; b, e: endpoint AUC from TCID_50_ measurements; c, f: endpoint temperature AUC; a, b, c: 50 patients in trial; d, e, f: 100 patients in trial.

a

b

c

d

f

e

Fig Q: Experiment 7: Frequency of endpoint measurements. Treatment acts on the virus production rate. Plots show the number of successful trials out of 100 runs (y-axis) over the assumed mean efficacy of treatment (x-axis). The probability of success corresponds to the power of the trial. The parameters determining the course of natural infection were drawn from the same random number distributions for each patient as explained in the main text. The efficacy for each patient (response) was fixed to the same value for each patient in each run. Thin coloured lines show the power of trial dependent on the efficacy of the treatment for different measurement protocols. “Roche” means the measurement protocol as described in the Methods section of the main text. The bold red line shows the power of the trial depending on the efficacy of the treatment when the simulated viral load curve is considered. Taking more than one viral load measurement per day does not increase the power of the trial. The same applies to temperature (symptom measurements). a, d endpoint viral load area under the curve (AUC) from qPCR measurements; b, e: endpoint AUC from TCID_50_ measurements; c, f: endpoint temperature AUC; a, b, c: 50 patients in trial; d, e, f: 100 patients in trial.

c

b

a

f

e

d

Fig R: Experiment 7: Frequency of endpoint measurements. Treatment acts on the virus clearance rate. Plots show the number of successful trials out of 100 runs (y-axis) over the assumed mean efficacy of treatment (x-axis). The probability of success corresponds to the power of the trial. The parameters determining the course of natural infection were drawn from the same random number distributions for each patient as explained in the main text. The efficacy for each patient (response) was fixed to the same value for each patient in each run. Thin coloured lines show the power of trial dependent on the efficacy of the treatment for different measurement protocols. “Roche” means the measurement protocol as described in the Methods section of the main text. The bold red line shows the power of the trial depending on the efficacy of the treatment when the simulated viral load curve is considered. Taking more than one viral load measurement per day does not increase the power of the trial. The same applies to temperature (symptom measurements a, d endpoint viral load area under the curve (AUC) from qPCR measurements; b, e: endpoint AUC from TCID_50_ measurements; c, f: endpoint temperature AUC; a, b, c: 50 patients in trial; d, e, f: 100 patients in trial.
